# Drug Transporters ABCB1 (P-gp) and OATP, but not Drug-Metabolizing Enzyme CYP3A4, Affect the Pharmacokinetics of the Psychoactive Alkaloid Ibogaine and its Metabolites

**DOI:** 10.3389/fphar.2022.855000

**Published:** 2022-03-04

**Authors:** Margarida L. F. Martins, Paniz Heydari, Wenlong Li, Alejandra Martínez-Chávez, Nikkie Venekamp, Maria C. Lebre, Luc Lucas, Jos H. Beijnen, Alfred H. Schinkel

**Affiliations:** ^1^ Division of Pharmacology, The Netherlands Cancer Institute, Amsterdam, Netherlands; ^2^ Department of Pharmacy and Pharmacology, The Netherlands Cancer Institute, Amsterdam, Netherlands; ^3^ Department of Pharmaceutical Sciences, Division of Pharmacoepidemiology and Clinical Pharmacology, Faculty of Science, Utrecht University, Utrecht, Netherlands

**Keywords:** ibogaine/iboga, noribogaine, P-glycoprotein/ABCB1, ABCG2/BCRP, OATP, CYP3A4, brain penetration, oral availability

## Abstract

The psychedelic alkaloid ibogaine is increasingly used as an oral treatment for substance use disorders, despite being unlicensed in most countries and having reported adverse events. Using wild-type and genetically modified mice, we investigated the impact of mouse (m)Abcb1a/1b and Abcg2 drug efflux transporters, human and mouse OATP drug uptake transporters, and the CYP3A drug-metabolizing complex on the pharmacokinetics of ibogaine and its main metabolites. Following oral ibogaine administration (10 mg/kg) to mice, we observed a rapid and extensive conversion of ibogaine to noribogaine (active metabolite) and noribogaine glucuronide. Mouse Abcb1a/1b, in combination with mAbcg2, modestly restricted the systemic exposure (plasma AUC) and peak plasma concentration (C_max_) of ibogaine. Accordingly, we found a ∼2-fold decrease in the relative recovery of ibogaine in the small intestine with fecal content in the absence of both transporters compared to the wild-type situation. Ibogaine presented good intrinsic brain penetration even in wild-type mice (brain-to-plasma ratio of 3.4). However, this was further increased by 1.5-fold in *Abcb1a/1b;Abcg2* ^*−/−*^ mice, but not in *Abcg2* ^*−/−*^ mice, revealing a stronger effect of mAbcb1a/1b in restricting ibogaine brain penetration. The studied human OATP transporters showed no major impact on ibogaine plasma and tissue disposition, but the mOatp1a/1b proteins modestly affected the plasma exposure of ibogaine metabolites and the tissue disposition of noribogaine glucuronide. No considerable role of mouse Cyp3a knockout or transgenic human CYP3A4 overexpression was observed in the pharmacokinetics of ibogaine and its metabolites. In summary, ABCB1, in combination with ABCG2, limits the oral availability of ibogaine, possibly by mediating its hepatobiliary and/or direct intestinal excretion. Moreover, ABCB1 restricts ibogaine brain penetration. Variation in ABCB1/ABCG2 activity due to genetic variation and/or pharmacologic inhibition might therefore affect ibogaine exposure in patients, but only to a limited extent. The insignificant impact of human CYP3A4 and OATP1B1/1B3 transporters may be clinically advantageous for ibogaine and noribogaine use, as it decreases the risks of undesirable drug interactions or interindividual variation related to CYP3A4 and/or OATP activity.

## Introduction

Ibogaine is a psychoactive indole alkaloid found in the root bark of the rainforest shrub *Tabernanthe iboga*, which is native to Central-West Africa (Alper, 2001; Mačiulaitis et al., 2008). Although controlled human clinical trials on the efficacy and safety of ibogaine are lacking, several studies, both in animals and in humans, report long-term drug abstinence from various substances, including opioids, alcohol, and psychostimulants, and sustained reductions in depressive symptoms after ibogaine administration (Mash et al., 2000; Schenberg et al., 2014; dos Santos et al., 2017; Brown and Alper, 2018; Mash, 2018; Mash et al., 2018 Wasko et al., 2018). Consequently, ibogaine, despite not being licensed as a therapeutic drug*,* has been increasingly used in informal self-help networks and treatment centers worldwide, mainly for the treatment of substance use disorders and for the management of opioid withdrawal symptoms (Brown, 2013; Brown and Alper, 2018; Corkery, 2018; Luz and Mash, 2021).

A major concern is the occurrence of possible adverse events related to the use of ibogaine, which include cardiac arrhythmias, psychosis, mania, seizures, and even fatalities (Koenig and Hilber, 2015; Litjens and Brunt, 2016; Schep et al., 2016; dos Santos et al., 2017; Wasko et al., 2018; Aćimović et al., 2021; Luz and Mash, 2021; Ona et al., 2021). However, most of these morbidities and mortalities occurred in uncontrolled/non-medical settings, where unknown or extremely high doses and variable purity of ibogaine were used (Alper et al., 2008; dos Santos et al., 2017). Otherwise, ibogaine seems to be reasonably safe when administered in more controlled/supervised contexts to individuals without previous cardiac complications or not under the acute effects of drugs (dos Santos et al., 2017).

**FIGURE 1:**
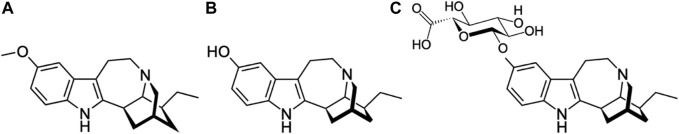
Molecular structures of ibogaine (MW: 310.4 g/mol) **(A)**, noribogaine (MW: 296.4 g/mol **(B)** and noribogaine glucuronide (MW: 471.5) **(C)**.

The main pharmacodynamically active metabolite of ibogaine in humans is noribogaine (O-desmethylibogaine or 12-hydroxyibogamine) (Glick et al., 2001; Mačiulaitis et al., 2008; Wasko et al., 2018), which subsequently undergoes glucuronidation (Glue et al., 2015a) (Figure 1). The metabolism of ibogaine occurs mainly in the liver but also in the gut, with CYP2D6 being the primary enzyme responsible for the conversion to noribogaine in humans (Obach et al., 1998; Mash et al., 2001; Glue et al., 2015b). It is worth noting that there is substantial variability in the CYP2D6 allele distribution among different ethnic groups (Bradford, 2002; Zhou, 2009), and many CYP2D6 substrates are subject to drug interactions (Monte et al., 2018). Thus, it may be wise to genotype patients expecting ibogaine treatment and to at least halve the intended dose of ibogaine in CYP2D6 poor metabolizers, as suggested by others (Obach et al., 1998; Mash et al., 2001; Glue et al., 2015b).

Moreover, there is *in vitro* evidence that CYP2C9 and CYP3A4 also contribute to some extent to ibogaine metabolism (Obach et al., 1998). In fact, conversion of the parent compound to noribogaine even in CYP2D6-deficient subjects may reflect the metabolic contribution of these other cytochromes (Mash et al., 2001). Nevertheless, further research should be undertaken to investigate the *in vivo* impact of those enzymes on the conversion of ibogaine to noribogaine. The subsequent conversion of noribogaine to noribogaine glucuronide is mainly mediated by glucuronosyltransferases (UGTs) (Glue et al., 2015a; 2015b; Glue et al., 2016).

Due to ibogaine lipophilicity, this alkaloid can accumulate in adipose tissue (Hough et al., 1996), which allows a slow release of ibogaine and subsequent rapid metabolism to noribogaine over an extended time period (Zubaran, 2000; Alper, 2001). Noribogaine, which displays a slow pharmacokinetic clearance (half-life of plasma noribogaine is around 24–30 h *versus* ibogaine 2–6 h) (Luz and Mash, 2021), is responsible for many of the effects seen after ibogaine therapy (Zubaran, 2000) and seems to be safe and well-tolerated when directly administered itself in healthy volunteers (Glue et al., 2015a). Ibogaine and noribogaine are excreted via urine and feces (60–70% of the administered dose in 24 h in rats) (Mačiulaitis et al., 2008).

The pharmacological targets responsible for the psychological and physiological actions of ibogaine and noribogaine are not entirely understood. Nevertheless, there is evidence suggesting that both ibogaine and noribogaine can interact, at the same time and with distinctive affinities, with multiple binding sites within the central nervous system (CNS), including, among others, dopamine and serotonin transporters, and N-methyl-D-aspartate (NMDA) and certain opioid receptors (Sweetnam et al., 1995; Glick and Maisonneuve, 1998; Alper, 2001; Mačiulaitis et al., 2008; Wasko et al., 2018). Thus, due to the slow release of ibogaine along with the complex pharmacodynamics of ibogaine and noribogaine, including their ability to modify addiction-related neural circuitry through the activation of neurotrophic factor signaling (He, 2005; Marton et al., 2019), these compounds can apparently elicit long-lasting and potentially irreversible behavioral and neurochemical changes in the brain of subjects, even after administration of a single ibogaine dose (Mash et al., 2018; Noller et al., 2018). Part of the success of ibogaine may also be explained by the creation of a profound dream-like experience after ibogaine administration that activates long-term memory, forcing a deep introspection that aids comprehension and resolution of conflicts related to the substance use disorder of the subject (Davis et al., 2018; Mash et al., 2018; Brown et al., 2019). Doses ranging from 4 to 29 mg/kg in the form of extracts/hydrochloride have been orally administered to achieve efficacy in patients pursuing release or at least interruption from drug dependency (Alper, 2001; dos Santos et al., 2017).

Currently, there are a few ongoing registered clinical studies involving ibogaine (hydrochloride) administration in the context of substance (namely opioids and alcohol) use disorder treatment (e.g., NCT04003948; NCT03380728; NCT05029401). In this context, and as the ibogaine subculture and the number of informal treatment centers continue to grow, it is imperative to investigate both pharmacokinetic and pharmacodynamic (PK-PD) aspects of ibogaine. Drug transporters and drug-metabolizing enzymes can modulate the PK-PD of drugs, which may provide new tools for improving drug safety and efficacy (Oesch, 2009; Giacomini et al., 2010; Li et al., 2019).

Most transmembrane drug transporters belong to one of two major families: the adenosine triphosphate (ATP)-binding cassette (ABC) superfamily and the organic anion transporting polypeptide (OATP) superfamily. P-glycoprotein (P-gp/MDR1/ABCB1) and breast cancer resistance protein (BCRP/ABCG2) are two important ABC drug efflux transporters that use the energy from ATP hydrolysis to translocate a great variety of substrates to the extracellular compartment (Giacomini et al., 2010). They are expressed throughout the body, including the luminal surface of brain capillary endothelial cells of the blood-brain barrier (BBB), where they actively pump certain drugs out of the brain (Ho and Kim, 2005; Giacomini et al., 2010). They can thus limit brain penetration and regulate the pharmacologic effects of their substrates. ABCB1 and ABCG2 are also expressed in the apical membrane of tissues with absorptive and eliminatory functions, such as the liver, intestine, and kidney. ABCB1 and ABCG2 in the bile canalicular membrane of hepatocytes facilitate the elimination of substrate drugs, pumping the compounds into the bile, while in the intestine, they can affect the net absorption of drugs as they excrete substrate drugs from the intestinal epithelium into the intestinal lumen. Finally, these ABC transporters are also expressed in the proximal tubules of nephrons, where they facilitate the efflux of substrate drugs from the blood into the urine (Ho and Kim, 2005; Giacomini et al., 2010).

OATPs/SLCOs are sodium-independent transmembrane uptake transporters (Ho and Kim, 2005; Giacomini et al., 2010). Owing to their localization in pharmacokinetically relevant tissues, such as the liver and kidney, and their broad substrate specificity, OATP1A and 1B transporters are thought to be instrumental in drug disposition (Kalliokoski and Niemi, 2009). OATP1A2 can be present in the apical membrane of the distal nephron, in the cells lining the bile ducts, and in the apical membrane of the brain capillary endothelial cells. OATP1B1 and OATP1B3 are highly expressed in the sinusoidal plasma membrane of hepatocytes, playing a central role in the hepatic uptake and plasma clearance of their drug substrates (Ho and Kim, 2005; Niemi, 2007; Iusuf et al., 2012).

Drug transporters can have pharmacological and toxicological effects by functioning alone or in combination with drug-metabolizing enzymes (Giacomini et al., 2010). Enzymes that belong to the Cytochrome P450 (CYP) superfamily mediate most phase I drug metabolism of various clinically used drugs and other xenobiotics (Benedetti et al., 2009; Li et al., 2019). Of all human CYPs, CYP3A4, which is mainly expressed in the liver and gastrointestinal tract, can metabolize more than 50% of the currently marketed drugs (Guengerich, 1999), affecting their bioavailability. Moreover, there are considerable inter- and intra-individual differences in CYP3A4 expression and activity, which can result from variable control of gene expression by endogenous molecules, or by drugs and food-derived xenobiotics, and from genetic polymorphisms (Lamba et al., 2002). Consequently, the oral availability and systemic clearance of CYP3A substrate drugs can differ widely among patients, which can instigate unexpected adverse events or therapeutic failures. This makes CYP3A4 one of the most relevant factors in variable drug exposure.

This study set out to assess the impact of the ABCB1 and ABCG2 drug efflux transporters and OATP drug uptake transporters on modulating oral availability and tissue distribution of ibogaine and its metabolites using wild-type and genetically modified mouse models. We also investigated the extent to which mouse and human CYP3A can have a role in the plasma exposure and tissue distribution of ibogaine *in vivo*.

## Materials and Methods

### Drugs, Chemicals, and Reagents

Ibogaine hydrochloride was acquired from Toronto Research Chemicals (North York, Canada), and noribogaine hydrochloride, noribogaine glucuronide lithium salt, and ibogaine-^13^C-d3 from TLC Pharmaceutical Standards (Newmarket, Canada). Distilled water was obtained from B. Braun Medical Supplies (Melsungen, Germany). Isoflurane was purchased from Virbac Nederland (Barneveld, The Netherlands), heparin (5000 IU ml^−1^) from Leo Pharma (Breda, The Netherlands), and bovine serum albumin (BSA, fraction V) from Roche Diagnostics GmbH (Mannheim, Germany). The chemicals used in the bioanalytical ibogaine assay were of ULC/MS grade and were obtained from Biosolve (Valkenswaard, The Netherlands), except for DMSO, which was purchased from Merck (Darmstadt, Germany). All other chemicals and reagents were obtained from Sigma-Aldrich (Steinheim, Germany).

### Animals

FVB wild-type (WT) and genetically modified strains were used, including *Abcg2* ^*−/−*^ (Jonker, 2000), *Abcb1a/1b;Abcg2* ^*−/−*^ (Jonker et al., 2005), *Oatp1a/1b* ^*−/−*^ (van de Steeg et al., 2010), *Oatp1a/1b* ^*−/−*^ *;1B1* ^*t*^ (van de Steeg et al., 2009), and *Oatp1a/1b* ^*−/−*^ *;1B3* ^*tg*^ (van de Steeg et al., 2013) (Oatp1a/1b-deficient mice with liver-specific expression of human OATP1B1 or OATP1B3), *Cyp3a* ^−/-^ and Cyp3aXAV (van Herwaarden et al., 2007) (transgenic mice with human (h)CYP3A4 expression in liver and small intestine in a *Cyp3a* ^−/-^ background).

Based on availability, female mice, all of a >99% FVB genetic background, were used between 9 and 15 weeks of age. Animals were kept in a temperature-controlled environment with a 12 h light/dark cycle. A standard diet (Transbreed, SDS Diets, Technilab-BMI, Someren, The Netherlands) and acidified water were provided to the mice *ad libitum.* Mice were housed and handled according to institutional guidelines in agreement with Dutch and EU Legislation (approval number from The Dutch Central Animal Testing Committee: AVD301002016595). The assessment of animal welfare was performed before and throughout the experiments.

### Drug Solutions

Ibogaine was dissolved in DMSO at a concentration of 10 mg/ml (stock solution) and stored at −70 C in an amber-colored falcon tube to avoid ibogaine degradation from daylight exposure, as suggested by other studies (Kontrimavičiut et al., 2005; Kontrimavičiute et al., 2006). On the day of the experiment, part of the stock solution was further diluted with saline (0.9% NaCl) to yield a concentration of 1 mg/ml of ibogaine.

### *In vivo* Pharmacokinetic Studies

To study the influence of drug uptake and efflux transporters on the pharmacokinetics of ibogaine and its metabolites, we conducted a 2 h experiment, while for the assessment of CYP3A impact, the experiments were terminated at 8 h. In each experiment, six to seven mice were used per strain. Ibogaine dissolved in DMSO:saline vehicle (1:9, v/v) was orally administered to the mice by gavage into the stomach, using a blunt-ended needle, at a single dose of 10 mg/kg (10 μL/g body weight). To reduce variation in absorption upon oral administration, mice were fasted for 2–3 h before dosing. Approximately 50 μL of blood was collected from the tip of the tail in heparinized capillary tubes (Sarstedt AG & Co., Nümbrecht, Germany) at 0.125, 0.25, 0.5 and 1 h (2 h experiment), or 0.25, 0.5, 1, 2, and 4 h (8 h experiment). For the 8 h experiment, 2 h after ibogaine administration, mice had *ad libitum* supply of standard chow and water again. At either 2 or 8 h after dosing, mice were deeply anesthetized with isoflurane (3%), followed by cardiac puncture to collect blood (at least 600 μL) in Eppendorf tubes using heparin as an anticoagulant. The mice were then sacrificed by cervical dislocation, and tissues including the brain, liver (without gallbladder), kidneys, spleen, small intestine together with the fecal content (SIWC), heart, and subcutaneous inguinal white adipose tissue were collected and weighed. For the 2 h experiment, the gallbladder with content was also (separately) collected in an Eppendorf tube and weighed. Plasma was obtained from blood samples by centrifugation at 9,000 *g* for 6 min at 4 °C. Tissue homogenates (except gallbladder) were obtained using the Fast Prep-24 5G homogenizer (MP Biomedicals Inc., Santa Ana, CA, United States). Before homogenization, Bovine Serum Albumin (BSA, Fraction V) was dissolved at a concentration of 2% (w/v) in distilled water. Three mL of ice-cold 2% BSA were added to liver and SIWC; 2 ml to kidneys; 1 ml to brain, spleen, and heart; and 2–3 ml to the white adipose tissue (depending on the tissue weight). For the gallbladder, 150 μL of 2% BSA was added; then, the samples were centrifuged (9,000 x *g*, 4°C, 6 min), and only the supernatant was collected for analysis. Both plasma and tissue samples were kept at −70°C in amber-colored Eppendorf tubes until analysis.

### LC-MS/MS Analysis

Ibogaine, noribogaine, and noribogaine glucuronide concentrations in plasma and tissue samples were determined using a liquid chromatography with tandem mass spectrometry (LC-MS/MS) method, validated for human plasma, mouse plasma, and mouse tissues. Since the concentration of ibogaine and its metabolites can rapidly decline at room temperature with daylight exposure, special attention was paid during sample handling to avoid photodegradation of the compounds.

A volume of 50 µL human plasma sample, a 5-fold diluted mouse plasma sample (10 uL + 40 uL control human plasma), or 100 µL homogenized tissue sample were processed. For the quantification, stable isotope labelled internal standards (IS) Ibogaine-^13^C-d3 (for ibogaine) and Noribogaine-d4 (for noribogaine and noribogaine glucuronide) were used. To the 50 uL plasma samples, 20 uL of a working solution containing both internal standards at a concentration of 10 ng/ml in methanol (WIS10) was added. Sample pre-treatment involved protein precipitation with 150 uL of acetonitrile-methanol (1:1, v/v). For the tissue homogenates (100 uL sample aliquots), 40 uL of WIS10 was added, and 300 uL precipitation reagent as sample cleanup. After centrifuging the samples (10 min, 4°C, 15,000 x *g*), 100 µL of the supernatant was transferred to an amber-colored autosampler vial with insert. Then 100 µL of 4 mM ammonium formate pH 3.5 in water was added, and after vortex mixing, the extracts were injected (5 uL) onto a UPLC system (Acquity UPLC I-Class IVD system, Waters, Milford, MA, United States) equipped with an Acquity UPLC HSS T3 Column (150 × 2.1 mm ID, 1.8 µm). The tray temperature was set to 5 ± 3°C, and the analytical column was kept at 50°C. Gradient elution was applied using 4 mM ammonium formate pH 3.5 in water as eluent A and acetonitrile as eluent B. The flow rate was 500 μL/min, and the total runtime was 6 min. Detection was performed in the positive ion mode on a Quadrupole MS/MS detector with a Turbo Ion Spray Interface (QTRAP5500, Sciex, Framingham, MA, United States). The ionspray voltage was 2250 V, and the spray temperature was 700 °C. Gas settings, potentials, and collision energies were optimized by infusion of the analytes and by flow injection analysis. The measured transitions were from m/z 311 to 174 for ibogaine, from m/z 297 to 122 for noribogaine, from m/z 473 to 297 noribogaine glucuronide, from m/z 315 to 178 for ibogaine-IS, and from m/z 301 to 122 for noribogaine-IS.

The method was validated for K_2_EDTA human plasma over the range from 0.1 to 50 ng/ml for ibogaine and 0.1–250 ng/ml for noribogaine and noribogaine glucuronide. Linear regression was used to fit the calibration lines, and a weighting factor of 1/conc^2^ was applied. Inter-assay accuracy and precision were within the required ±15%. At the lower limit of quantification (LLOQ), the signal-to-noise was at least 5 for three consecutive analytical runs for all analytes. Carry-over was acceptable (≤20% of the LLOQ in the first blank after injecting an upper limit of quantification (ULOQ) sample).

Human ibogaine plasma samples could be diluted in 0.2 or 2% BSA, and dilutions factors of 10 and 100 times were validated. The analytes are stable in the stock solutions (1 mg/ml in DMSO) for at least 340 days, in the working solutions (10,000 ng/ml and 2.00 ng/ml in methanol) for at least 419 and 153 days, respectively, when stored at −70°C. Stability was demonstrated in the biomatrix (K_2_EDTA human plasma) after 3 freeze (−70°C)/thaw (room temperature) cycles, at room temperature for at least 72 h when exposed to light and in the dark, and 258 days when stored at−70°C. The final extracts were stable for at least 4 days when stored at 2–8°C.

Validation tests were executed to investigate whether calibration standards prepared in control human plasma could be used for the quantification of the analytes in mouse plasma and mouse tissues. Since no stable isotope internal standard was commercially available for noribogaine glucuronide, a ±20% requirement for the bias was applied for noribogaine glucuronide in plasma (instead of 15%) and 25% in tissues (instead of 20%). Mice plasma samples (originating from knockout and wild-type mice) were spiked with 187.5 ng/ml of each analyte, diluted 5 times in control human plasma, and subsequently processed and quantified (*n* = 5). Accuracy and precision values were within the requirements. Furthermore, blank tissue homogenates were spiked with the analytes at a concentration of 37.5 ng/ml (*n* = 1). Samples were processed and quantified (for the quantification of noribogaine glucuronide in brain, kidney, and heart, ibogaine-IS was used), and all bias values were within the pre-defined requirements, except for kidney (ibogaine and noribogaine) and gallbladder (noribogaine) samples. Ion suppression in these samples was considerable, and the internal standards were not capable of correcting for these matrix effects. Deviations from the nominal concentration in these two matrices were between −38.1 and −24.3%.

### Pharmacokinetic Calculations and Statistical Analysis

Pharmacokinetic parameters were calculated by non-compartmental methods using the Microsoft Excel menu-driven add-in program PKsolver (Zhang et al., 2010). The area under the plasma concentration-time curve (AUC) was calculated with the trapezoidal rule without extrapolating to infinity. The peak plasma concentration (C_max_) and the time to reach C_max_ (T_max_) were estimated from the original data. The tissue-to-plasma ratios of ibogaine and its metabolites were calculated by dividing the tissue drug concentration by the plasma concentration at the terminal time point, while tissue accumulation was calculated by determining the drug tissue concentration relative to its plasma AUC from 0 to 2 h or 0–8 h. GraphPad Prism 9 (GraphPad Software Inc., San Diego, CA, United States) was used to plot the data and to perform statistical analysis. To determine whether the most extreme value in a data set was a significant outlier from the rest, the Grubbs’s (or extreme studentized variate) test was applied (α = 0.05). All data were log-transformed before statistical analysis. Ordinary one-way analysis of variance (ANOVA) was used when multiple groups were compared, and the appropriate post hoc correction was applied to accommodate multiple testing. Differences were considered statistically significant when *p* < 0.05. All data are presented as mean ± SD.

## Results

### ABCB1 in Combination With ABCG2 Facilitates Ibogaine Plasma Clearance

In order to assess the single and combined effects of the drug efflux transporters Abcb1 and Abcg2 on the plasma pharmacokinetics and tissue disposition of ibogaine and its pharmacodynamically active metabolite noribogaine, and noribogaine glucuronide, we conducted a 2-h study using female WT, *Abcg2* ^*−/−*^, and *Abcb1a/1b;Abcg2* ^*−/−*^ mice. Because ibogaine is usually taken orally by patients (4–29 mg/kg), we administered ibogaine orally to mice at a dose of 10 mg/kg.

**FIGURE 2:**
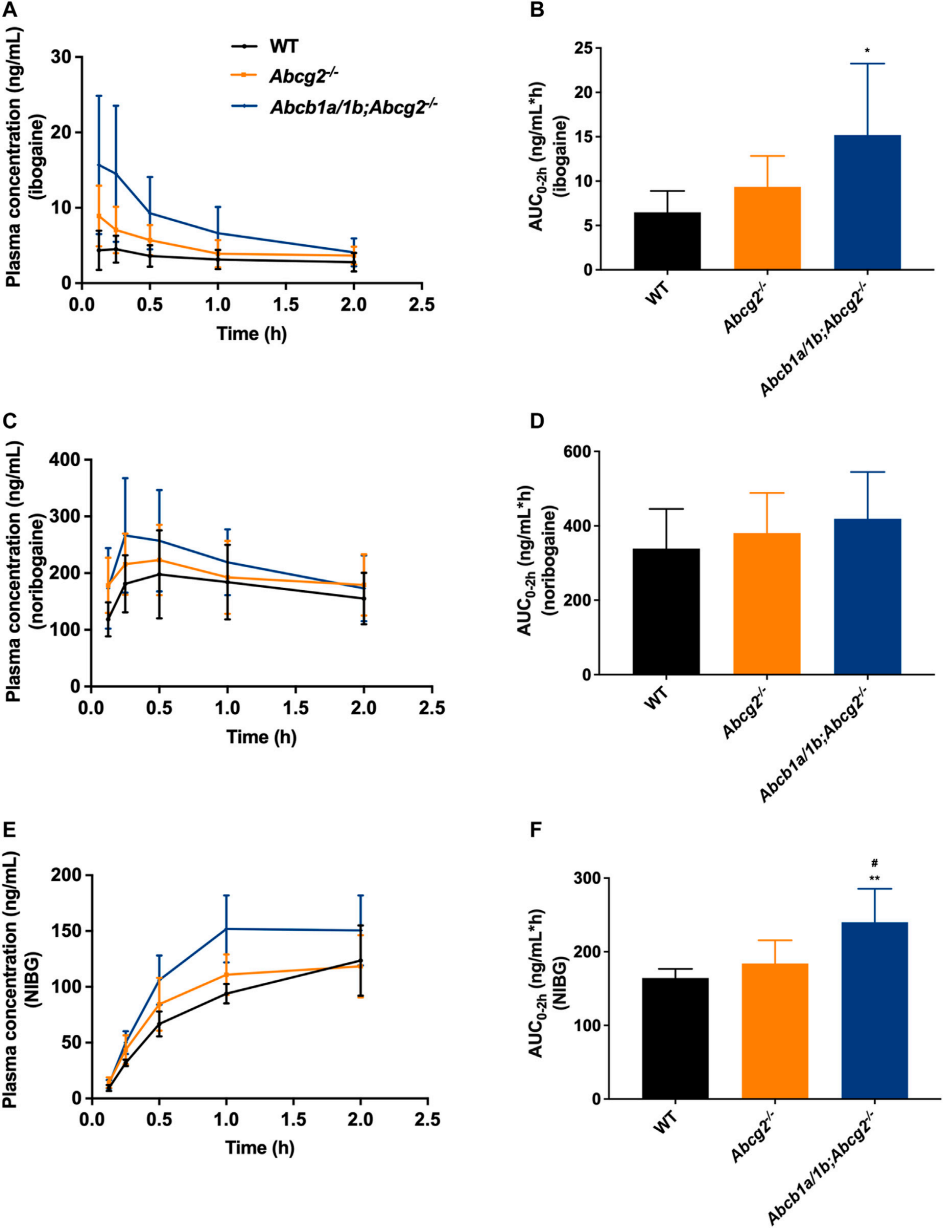
Plasma concentration-time curves and area under the plasma concentration-time curves (AUC) of ibogaine **(A,B)**, noribogaine **(C,D)**, and noribogaine glucuronide (NIBG) **(E,F)**, respectively, in female wild-type (WT), *Abcg2* ^*−/−*^, and *Abcb1a/1b;Abcg2* ^*−/−*^ mice, over 2 h after oral administration of 10 mg/kg ibogaine (*n* = 7). Data are presented as mean ± SD. ^*^, *p* < 0.05; ^**^, *p* < 0.01 compared to wild-type mice; ^#^, *p* < 0.05 comparing *Abcb1a/1b;Abcg2* ^*−/−*^ with *Abcg2* ^*−/−*^ mice.

Oral absorption of ibogaine in mice was very rapid, with the highest plasma concentrations generally observed well within half an hour after dosing (Figure 2; Table 1). Moreover, as in humans, there was an extensive and rapid conversion of ibogaine to noribogaine and noribogaine glucuronide, likely due to first-pass metabolism effects in the small intestine and liver. Maximal concentrations and AUCs of the metabolites in plasma actually far exceeded those of the parent compound (Figure 2; Table 1).

**TABLE 1:** Plasma pharmacokinetic (PK) parameters of ibogaine, noribogaine, and noribogaine glucuronide 2 h after oral administration of 10 mg/kg ibogaine to female wild-type, *Abcg2* ^*−/−*^, *and Abcb1a/1b;Abcg2* ^*−/−*^ mice^a^.

Compound and PK Parameter	Genotype		
	Wild-type	Abcg2^−/−^	Abcb1a/1b; Abcg2^−/−^
Ibogaine			
AUC_0-2h_ (h*ng/mL)	6.48 ± 2.40	9.35 ± 3.47	15.2 ± 8.06^*^
Fold increase AUC_0-2h_	1.0	1.4	2.3
C_max_, ng/mL	4.92 ± 2.34	9.15 ± 3.69	15.92 ± 9.16^**^
T_max_, h	0.25 (0.125–2)	0.125 (0.125–0.50)	0.25 (0.125–0.25)
Noribogaine			
AUC_0-2h_ (h*ng/mL)	339 ± 107	381 ± 108	419 ± 126
Fold increase AUC_0-2h_	1.0	1.1	1.2
C_max_, ng/mL	208 ± 70	233 ± 61	276 ± 98
T_max_, h	0.50 (0.25–1)	0.25 (0.125–0.50)	0.25 (0.25–2)
Noribogaine glucuronide			
AUC_0-2h_ (h*ng/mL)	164 ± 12	184 ± 32	240 ± 46^**^ ^/^ ^#^
Fold increase AUC_0-2h_	1.0	1.1	1.5
C_max_, ng/mL	125 ± 30	122 ± 27	156 ± 31
T_max_, h	2 (1–2)	2 (1–2)	2 (1–2)

a Data are presented as mean ± SD (*n* = 7), except for T_max_ where median (range) is presented. AUC_0–2h_, area under the plasma concentration-time curve from zero to 2 h; C_max_, maximum concentration in plasma; T_max_, time point (h) of maximum plasma concentration.^*^, *p* < 0.05; ^**^, *p* < 0.01 compared to wild-type mice; ^#^, *p* < 0.05 comparing *Abcb1a/1b;Abcg2* ^*−/−*^ with *Abcg2* ^*−/−*^ mice.

The area under the plasma concentration-time curves (AUC_0–2h_) revealed a modest but significant increase in ibogaine oral availability between WT and *Abcb1a/1b;Abcg2* ^*−/−*^ mice, but not for *Abcg2* ^*−/−*^ mice (Figure 2; Table 1). This suggests that the mouse (m)Abcb1a/1b transporter plays a significant role in ibogaine clearance and perhaps in limiting its absorption; thus, when absent, more ibogaine is available systemically, as the AUC significantly increased by 2.3-fold compared to WT mice (Figure 2; Table 1). For noribogaine, the studied drug efflux transporters had no significant impact on its systemic availability and elimination (Figure 2; Table 1). The plasma exposure of noribogaine glucuronide over 2 h (AUC_0–2h_) was slightly but significantly increased by the combined absence of Abcb1a/1b and Abcg2 (1.5-fold) compared to WT mice, but not by the lack of Abcg2 alone (Figure 2; Table 1).

In summary, the plasma exposure of ibogaine and noribogaine glucuronide was modestly but significantly restricted by Abcb1a/1b and perhaps Abcg2, whereas the noribogaine absorption profile showed a similar but non-significant trend. Because the NIBG/NIB ratio for the AUCs was not statistically significantly different among the studied mouse strains (data not shown), it seems likely that the higher C_max_ and AUC values for noribogaine glucuronide simply reflected the somewhat higher noribogaine plasma concentrations that we observed.

### ABCB1 and ABCG2 Limit Brain and Kidney Levels of Ibogaine and Contribute to its Small Intestinal Disposition

We also measured the concentration of ibogaine and its metabolites in the brain, SIWC, liver, gallbladder, spleen, kidneys, heart, and inguinal white adipose tissue 2 h after ibogaine oral administration to mice.

**FIGURE 3:**
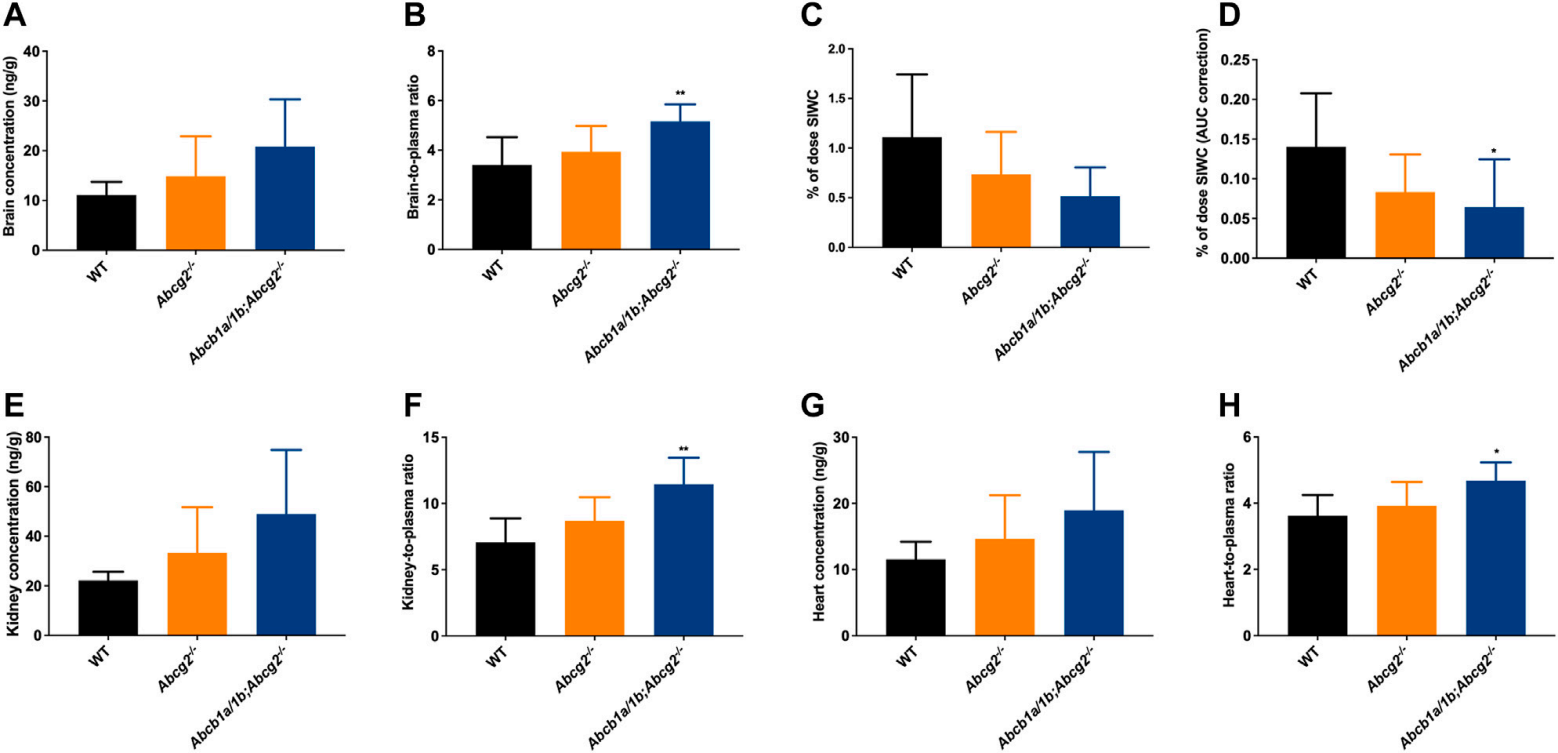
Ibogaine brain, kidney and heart concentrations **(A,E,G)**, tissue-to-plasma ratios **(B,F,H)**, and small intestine together with the fecal content (SIWC) as percentage of dose **(C)** corrected for the plasma AUC **(D)** in female wild-type (WT), *Abcg2* ^*−/−*^, and *Abcb1a/1b;Abcg2* ^*−/−*^ mice, over 2 h after oral administration of 10 mg/kg ibogaine (*n* = 6–7). Data are presented as mean ± SD. ^*^, *p* < 0.05; ^**^, *p* < 0.01 compared to wild-type mice.

Although no significant differences were observed between the studied mouse strains regarding ibogaine brain concentrations due to high interindividual variation (Figure 3A), we did find a significantly higher ibogaine brain-to-plasma ratio in *Abcb1a/1b;Abcg2* ^*−/−*^ mice compared to WT mice (1.5-fold increase, Figure 3B). This suggests that when mAbcb1a and mAbcg2 are absent, there is less ibogaine efflux from the brain into the systemic circulation; thus, relatively more ibogaine is present in the brain tissue, on top of its higher plasma exposure.

We further observed a significant 2.2-fold decrease in the total ibogaine amount (% of dose) in the SIWC corrected for the plasma exposure (AUC) over 2 h in *Abcb1a/1b;Abcg2* ^*−/−*^ mice compared to WT mice (Figure 3D). This indicates that mAbcb1 and perhaps mAbcg2 have a significant impact on either directly or indirectly excreting ibogaine towards the small intestine lumen or on keeping it in the intestinal lumen or both. Notably, the liver and spleen disposition of ibogaine (Supplementary Figures S2A, B, E, F) was not increased in the Abcb1a/1b; Abcg2-deficient mice. Moreover, there was no or very little active excretion of ibogaine from the liver into the bile, considering the very low gallbladder distribution of this compound (Supplementary Figures S2C, D).

Concerning the kidney, we observed a significant 1.6-fold increase in tissue-to-plasma ratio in *Abcb1a/1b;Abcg2* ^*−/−*^ mice compared to WT mice (Figure 3F). This might be due to the reduced elimination of ibogaine from the kidney, normally mediated by the studied ABC transporters. We also observed an increase in the heart-to-plasma ratio when both mAbcb1a/1b and mAbcg2 were ablated compared to the WT situation, although the shift, while significant, was very modest (1.3-fold increase; Figure 3H). For the other analyzed tissues, no meaningful differences were found regarding ibogaine disposition, except for the SIWC values as explained above (Supplementary Figure S2).

For noribogaine, we did not observe a significant impact of the studied efflux transporters on its tissue disposition in any of the analyzed tissues or compartments (Supplementary Figure S3). Remarkably, the ibogaine and noribogaine brain-to-plasma ratios (Figure 3B; Supplementary Figure S3B) among the studied mouse strains were in the same order for these two compounds, even though the noribogaine plasma exposure was far higher than that of ibogaine. Noribogaine also appeared not to be substantially concentrated in the bile, as the liver-to-plasma ratios were at least 3 times higher than the gallbladder-to-plasma ratios (Supplementary Figures S3D, F).

Noribogaine glucuronide distribution to most tissues was not significantly affected by the ABC transporter deficiencies (Supplementary Figure S4), generally following the somewhat increased plasma concentration of this compound in *Abcb1a/1b;Abcg2* ^*−/−*^ mice (Figures 2E,F). However, we observed a 1.7-fold decrease in the liver-to-plasma ratio in *Abcb1a/1b;Abcg2* ^*−/−*^ mice compared to the WT strain (Supplementary Figure S4D). The gallbladder-to-plasma ratios of the glucuronide were 5- to 10-fold higher than entire liver-to-plasma ratios, suggesting a marked concentration of the glucuronide in the bile (Supplementary Figure S4F). They were also more than 2-fold lower in *Abcb1a/1b;Abcg2* ^*−/−*^ than in WT mice, although this was not statistically significant. If this situation would also apply to intrahepatic bile, this might in part explain the significantly reduced whole liver-to-plasma ratios of the glucuronide in *Abcb1a/1b;Abcg2* ^*−/−*^ mice. Moreover, we found a modest but significant decrease in the total amount (% of dose) of noribogaine glucuronide corrected for the plasma exposure (AUC) over 2 h in the SIWC in *Abcg2* ^*−/−*^ (1.5-fold decrease) and *Abcb1a/1b;Abcg2* ^*−/−*^ mice (2.1-fold decrease) compared to the WT situation (Supplementary Figures S4Q−S). Therefore, these findings suggest that these ABC transporters mediate glucuronide excretion into the intestinal lumen either through biliary and/or direct intestinal excretion.

The brain-to-plasma ratios of noribogaine glucuronide were much lower (50- to 100-fold) than those of ibogaine and noribogaine (Figure 3B; Supplementary Figures S3B, S4B), indicating very low brain penetration. This was not unforeseen since noribogaine glucuronide is a much more polar compound than noribogaine and ibogaine.

Moreover, we observed considerably higher adipose tissue-to-plasma distribution ratios for ibogaine. This was expected, as this compound is more lipophilic compared with its metabolites (ratios in WT mice of 45.3 (ibogaine) *versus* 2.8 (noribogaine) *versus* 0.058 (noribogaine glucuronide); Supplementary Figures S2H, S3N, S4N).

Collectively, our findings suggest that mAbcb1 in combination with mAbcg2 can restrict brain, kidney, and perhaps heart penetration of ibogaine and possibly perform its hepatobiliary and/or direct intestinal excretion. On the other hand, the studied ABC transporters have no meaningful impact on noribogaine tissue disposition. For noribogaine glucuronide, the absence of mAbcb1 in the apical surface of hepatocytes results in lower liver disposition, while the absence of both mAbcb1 and mAbcg2 yields a decrease in the total amount of this metabolite recovered in the SIWC.

### Effect of Mouse Oatp1a/1b, but Not Human OATP1B1/1B3 on Plasma Pharmacokinetics and Tissue Disposition of Oral Ibogaine and Its Metabolites

It is known that ibogaine metabolism mainly occurs in the liver; still, how ibogaine enters this tissue is not entirely understood. In order to study the poorly elucidated interactions of ibogaine and its metabolites with OATP transporters, we administered ibogaine orally (10 mg/kg) to female WT, *Oatp1a/1b* ^*−/−*^ *, Oatp1a/1b* ^*−/−*^ *;1B1* ^*tg*^, and *Oatp1a/1b* ^*−/−*^ *;1B3* ^*tg*^ mouse strains.

**FIGURE 4:**
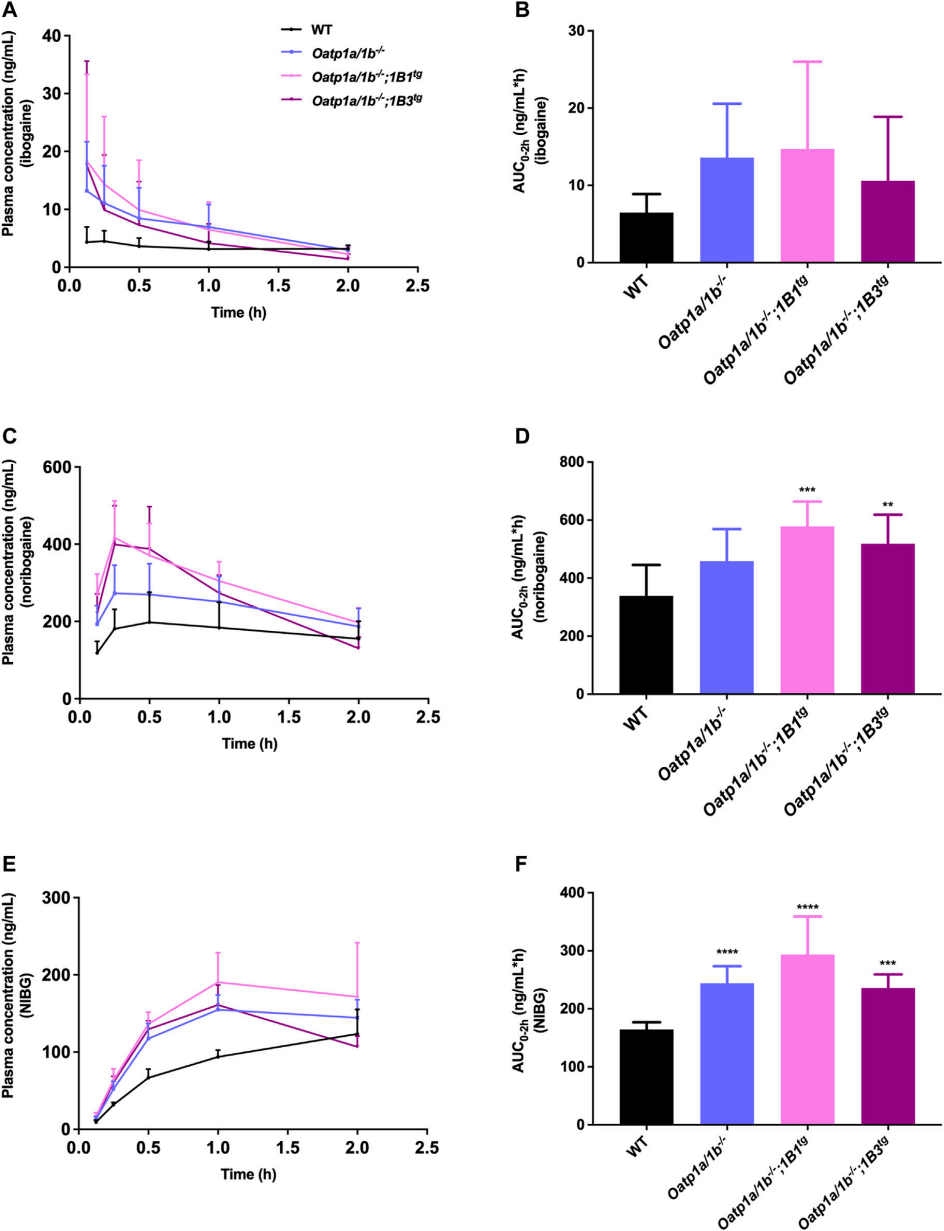
Plasma concentration-time curves and plasma AUCs of ibogaine **(A,B)**, noribogaine **(C,D)**, and noribogaine glucuronide (NIBG) **(E,F)**, respectively, in female wild-type (WT), *Oatp1a/1b* ^*−/−*^, *Oatp1a/1b* ^*−/−*^ *;1B1* ^*tg*^, and *Oatp1a/1b* ^*−/−*^ *;1B3* ^*tg*^ mice, over 2 h after oral administration of 10 mg/kg ibogaine (*n* = 7). Data are presented as mean ± SD.^**^, *p* < 0.01; ^***^, *p* < 0.001; ^****^, *p* < 0.0001 compared to wild-type mice.

First, we analyzed the plasma concentrations up to 2 h of ibogaine and its metabolites. The ibogaine plasma AUC_0–2h_ was not significantly different between the studied mouse strains due to high interindividual variation, although there was a tendency for higher levels in all the Oatp1a/1b-deficient strains (Figure 4; Table 2). However, for noribogaine, we observed a significantly higher plasma exposure in *Oatp1a/1b* ^*−/−*^ *;1B1* ^*tg*^ and *Oatp1a/1b* ^*−/−*^ *;1B3* ^*tg*^ mice compared to the WT situation (Figures 4C,D; Table 2), while for noribogaine glucuronide, the plasma exposure was considerably higher in all Oatp1a/1b-deficient mouse strains compared to WT mice (Figures 4E,F; Table 2). This suggests that mOatp1a/1b contributed to the elimination of noribogaine and noribogaine glucuronide from plasma; thus, when these transporters are absent, there is more metabolite available in the systemic circulation. Nevertheless, when transgenic human OATPs are present, one would expect reduced plasma exposure of their transported substrates compared to *Oatp1a/1b* ^*−/−*^ mice due to a more efficient tissue uptake, especially into the liver. Our findings, therefore, indicate that the human OATP1B1 and 1B3 proteins have little, if any, impact on the oral availability of ibogaine and on the elimination from plasma of its metabolites in mice.

**TABLE 2:** Plasma pharmacokinetic (PK) parameters of ibogaine, noribogaine, and noribogaine glucuronide 2 h after oral administration of 10 mg/kg ibogaine to female wild-type, *Oatp1a/1b* ^*−/−*^, *Oatp1a/1b* ^*−/−*^ *;1B1* ^*tg*^, and *Oatp1a/1b* ^*−/−*^ *;1B3* ^*tg*^ mice^a^.

Compound and PK Parameter	Genotype			
	Wild-type	Oatp1a/1b^−/−^	Oatp1a/1b^−/−^;1B1^tg^	Oatp1a/1b^−/−^;1B3^tg^
Ibogaine				
AUC_0-2h_ (h*ng/mL)	6.48 ± 2.40	13.6 ± 6.97	14.7 ± 11.3	10.6 ± 8.27
Fold increase AUC_0-2h_	1.0	2.1	2.3	1.6
C_max_, ng/mL	4.92 ± 2.34	13.6 ± 8.6	18.3 ± 15.0^*^	18.0 ± 17.7
T_max_, h	0.25 (0.125–2)	0.125 (0.125–0.50)	0.125	0.125 (0.125–0.25)
Noribogaine				
AUC_0-2h_ (h*ng/mL)	339 ± 107	458 ± 110	578 ± 86^***^	519 ± 100^**^
Fold increase AUC_0-2h_	1.0	1.4	1.7	1.5
C_max_, ng/mL	209 ± 70	290 ± 73	417 ± 95^***^	411 ± 107^***^
T_max_, h	0.50 (0.25–1)	0.50 (0.25–1)	0.25	0.25 (0.25–0.50)
Noribogaine glucuronide				
AUC_0-2h_ (h*ng/mL)	164 ± 12	244 ± 30^****^	294 ± 65^****^	236 ± 24^***^
Fold increase AUC_0-2h_	1.0	1.5	1.8	1.4
C_max_, ng/mL	125 ± 30	155 ± 20	199 ± 59^**^	162 ± 25
T_max_, h	2 (1–2)	2 (1–2)	1 (1–2)	1

a Data are presented as mean ± SD (n = 7), except for T_max_ where median (range) is presented. AUC_0–2h_, area under the plasma concentration-time curve from zero to 2 h; C_max_, maximum concentration in plasma; T_max_, time point (h) of maximum plasma concentration. ^**^, *p* < 0.01; ^***^, *p* < 0.001; ^****^, *p* < 0.0001 compared to wild-type mice.

We next evaluated the tissue distribution of ibogaine and its metabolites at 2 h. As the plasma concentrations of ibogaine between the strains at the termination time point were somewhat erratic, and in part virtually inversed from the plasma AUC order (Figure 4A; WT highest plasma concentration at 2 h, but lowest AUC_0–2h_), we primarily focused on the assessment of the tissue accumulation data, also as ibogaine tends to accumulate in various tissues. The most striking effect was observed for the liver accumulation, with some reduction (>2-fold) seen in *Oatp1a/1b* ^*−/−*^ mice, albeit not significant, but a very marked reduction in both transgenic strains (Supplementary Figure S5B). The most likely explanation for this observation is that mOatp1a/1b is partly responsible for the liver uptake and thus clearance of ibogaine, but that experimental variation obscured this effect in the *Oatp1a/1b* ^*−/−*^ mice, whereas the human OATP1B1 and 1B3 could not compensate for this absence of function in the transgenic strains. Somewhat similar trends were observed in the heart, spleen, and brain, although much less outspoken than for the liver.

Also, for noribogaine, the plasma concentration curves were somewhat erratic between the strains when comparing plasma AUC_0–2h_ with the plasma concentration at 2 h (Figure 4C), so we likewise focused on the analysis of the tissue accumulation data. The data suggest that noribogaine may be transported by mOatp1a/1b based on its liver accumulation (Supplementary Figure S6B), which was modestly decreased in *Oatp1a/1b* ^*−/−*^ and the two transgenic strains mice compared to WT mice (1.6-, 1.8-, and 2.1-fold, respectively). Evidently, human OATP1B1 and 1B3 could not compensate for the loss of mOatp1a/1b in the liver. Spleen accumulation showed a similar profile as liver, and to a lesser extent, heart. Likewise, the SIWC, whether expressed as accumulation or % of dose corrected for AUC, showed similar behavior.

Concerning noribogaine glucuronide, we observed a profile indicating clearly less liver accumulation, a strongly reduced gallbladder concentration and accumulation, and also markedly reduced accumulation and % of dose recovered in the SIWC of all Oatp1a/1b-deficient mouse strains compared to the WT situation (Supplementary Figure S7). The reduced liver accumulation likely secondarily causes a strongly reduced bile concentration and hence hepatobiliary excretion into the small intestinal lumen, explaining the reduced SIWC values. Thus, this metabolite is likely efficiently transported by hepatic mOatp1a/1b transporters, but apparently not by the human OATP1B1 and 1B3. It also appears to be strongly concentrated in the bile (WT gallbladder-to-plasma ratio about 10-fold higher than liver-to-plasma ratio), in contrast to ibogaine and noribogaine.

In summary, our results suggest that mOatp1a/1b plays a role in the liver uptake of ibogaine metabolites, and perhaps ibogaine itself, and contributes to the intestinal excretion of the metabolites, and thus clearance of these compounds. In contrast, human OATP1B1 and 1B3 do not seem to impact the plasma clearance and tissue disposition of ibogaine and its metabolites.

### Limited *in vivo* Impact of Cyp3a on Ibogaine Pharmacokinetics

**FIGURE 5:**
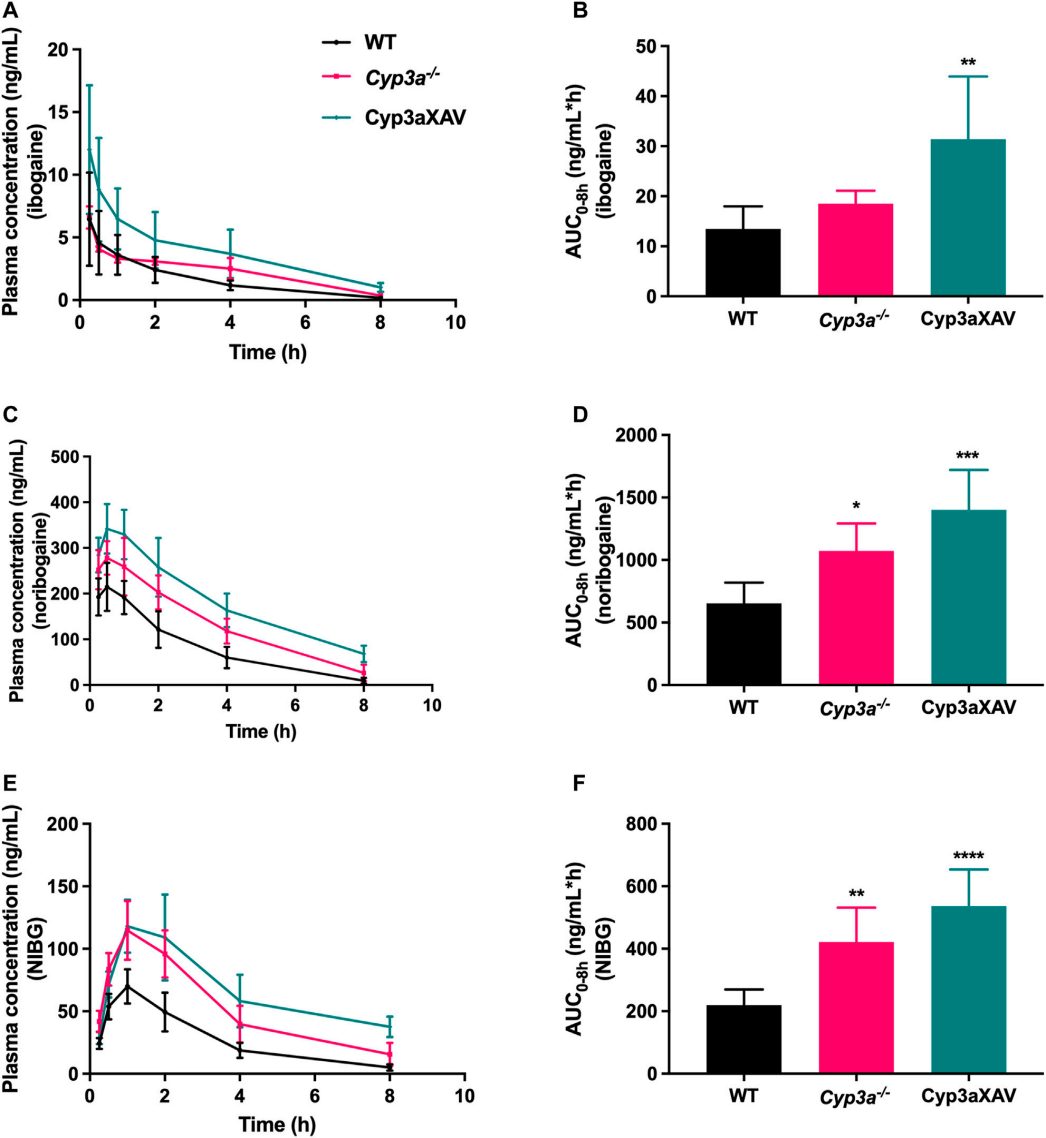
Plasma concentration-time curves **(A,C,E)** and plasma AUCs (AUC_0–8h_) **(B,D,F)** of ibogaine, noribogaine, and noribogaine glucuronide (NIBG) in female wild-type (WT), Cyp3a knockout (*Cyp3a* ^*−/−*^), and *Cyp3a* ^*−/−*^ mice with specific transgenic expression of human CYP3A4 in liver and intestine (Cyp3aXAV), over 8 h after oral administration of 10 mg/kg ibogaine (*n* = 4–6). Data are presented as mean ± SD. ^*^, *p* < 0.05; ^**^, *p* < 0.01; ^***^, *p* < 0.001; ^****^, *p* < 0.0001 compared to wild-type mice.

To date, there is evidence only from *in vitro* studies suggesting a minor contribution of the CYP3A4 enzyme to the conversion of ibogaine to noribogaine. To study the possible *in vivo* roles of mouse (m)Cyp3a and human CYP3A4 in ibogaine metabolism and thus pharmacokinetics, female WT, Cyp3a knockout (*Cyp3a* ^*−/−*^), and *Cyp3a* ^*−/−*^ mice with specific transgenic expression of human CYP3A4 in liver and intestine (Cyp3aXAV) were used in an 8 h experiment. After oral administration of 10 mg/kg ibogaine, blood and tissues were collected and processed as described above. As observed in the previous drug transporter experiments, the concentrations of the metabolites in plasma far exceeded those of the parent compound in all strains. Moreover, the concentration levels of ibogaine and its metabolites considerably declined over 8 h, especially for the parent compound (Figure 5; Supplementary Table S1).

Considering the impact of mCyp3a and human CYP3A4 on ibogaine and its metabolites, the plasma results were quite complex and partly counterintuitive. It seems unlikely that mCyp3a or human CYP3A4 is significantly involved in the conversion from ibogaine to noribogaine, as this would predict a relative increase in ibogaine and a decrease in noribogaine in the absence of these enzymes. What we observed instead was a modest increase of both compounds in the *Cyp3a* ^*−/−*^ compared to WT mice, albeit only significant for noribogaine (Figures 5A–D). This, in theory, could be explained if mCyp3a would modestly convert both ibogaine and noribogaine to other metabolites that we do not detect. However, if human CYP3A4 would catalyze the same reactions, this should result in a decrease of both compounds in Cyp3aXAV compared to *Cyp3a* ^*−/−*^ mice. Instead, the concentrations of both compounds rose even further, albeit relatively modestly and not statistically significantly (Figures 5A–D). This suggests that CYP3A4 does not noticeably metabolize ibogaine or noribogaine *in vivo*. Also, the noribogaine-to-ibogaine plasma AUC ratios were similar between the strains (data not shown), consistent with the absence of CYP3A-mediated conversion from ibogaine to noribogaine.

Noribogaine glucuronide plasma concentrations mostly followed the profile of noribogaine, and therefore likely just reflected the concentration changes of noribogaine (Figures 5C–F). This stands to reason, as most likely a UGT enzyme and not CYP3A is involved in this conversion.

The tissue distribution data for ibogaine itself were again quite complicated, even if the absolute concentrations were only a fraction of those of its metabolites in most tissues, often only a few percent of noribogaine values (Supplementary Figure S8). The general pattern was one of progressively increased tissue concentrations and tissue-to-plasma ratios of ibogaine going from WT to *Cyp3a* ^*−/−*^ and then Cyp3aXAV mice, with only liver-to-plasma and SIWC-to-plasma ratios showing little difference between the strains albeit with high experimental variation. The underlying mechanism is unclear, although reduced elimination of ibogaine in the two Cyp3a-deficient mouse strains may play a role. As also observed in the drug transporter studies, the ibogaine tissue-to-plasma ratios suggest that ibogaine can accumulate in adipose tissue, where its levels become much higher than in plasma (Supplementary Figure S8N).

Noribogaine tissue concentrations followed the plasma concentrations in most tissues judging from the tissue-to-plasma ratios (Supplementary Figure S9), with perhaps the liver as an exception with significantly reduced tissue-to-plasma ratios in the knockout and transgenic strains. Theoretically, this might happen if hepatic (re-)uptake of noribogaine was reduced in both of these strains, which would also fit with the higher plasma concentrations and AUCs of noribogaine observed. Overall, it seems that noribogaine can easily enter all the tissues, having a large volume of distribution, as is also the case in humans. Nevertheless, the plasma concentration of noribogaine is much higher than for ibogaine, which may facilitate easy distribution to tissues. The exception was the noribogaine disposition in adipose tissue, which was lower than for ibogaine. This was expected since the metabolite is a more polar compound (Supplementary Figure S9N).

Once again, like the plasma levels, tissue levels of noribogaine glucuronide mostly reflected those of its precursor noribogaine (Supplementary Figure S10). A possible exception was the SIWC-to-plasma ratio, which most likely reflected the different disposition of the glucuronide in liver as a consequence of biliary excretion of this compound.

Given the pharmacodynamic and therapeutic role(s) of ibogaine and noribogaine in the brain, it is noteworthy that the absolute brain concentrations of ibogaine and noribogaine were strongly increased in *Cyp3a* ^*−/−*^ and especially Cyp3aXAV mice (Supplementary Figures S8A, S9A). A similar shift was found in the heart (Supplementary Figures S8K, S9K). Nonetheless, in spite of the increased brain and heart concentrations, no abnormal behavior or signs of acute toxicity (such as tremors or flat body posture) were detected after oral administration of 10 mg/kg ibogaine during the whole experiment (8 h) among the studied mouse strains. The same holds true for the 2 h experiments, where the impact of drug efflux and uptake transporters was assessed. It is further worth noting that the absolute brain concentrations of noribogaine were far higher (at least ∼100-fold in wild-type mice) than those of ibogaine, both at 2 and 8 h.

In short, the findings obtained from the drug-metabolizing enzymes study indicate that ablation of mouse Cyp3a or overexpression of human CYP3A4 did not directly lead to the anticipated increase or decrease, respectively, of ibogaine availability, assuming that CYP3A would substantially metabolize ibogaine in mice. Instead, the data indicate that there is a secondary modulation or compensatory function of one or more other ibogaine uptake or detoxification system(s) not yet identified when CYP3A is removed or reintroduced. This could then account for the modest (and often counterintuitive) pharmacokinetic differences that we found between the studied mouse strains.

## Discussion

Interest in ibogaine for its potential antiaddictive properties has been gaining momentum in the past decades. Nevertheless, research to date on the interactions of ibogaine and its metabolites with drug transporters and metabolizing enzymes has been very limited. This is a drawback as a person with substance use disorder may use all sorts of medications, including antiretroviral, antipsychotic, antidepressant, and anxiolytic drugs that can be substrates or inducers/inhibitors of the same transporters/enzymes, thus resulting, for instance, in detrimental drug-drug interactions.

As in humans, we found that ibogaine was rapidly absorbed and quickly and extensively converted to noribogaine, which subsequently underwent glucuronidation. The low ibogaine plasma concentrations modestly decreased over time, which made it challenging to detect, especially at later time points. This suggests that the parent compound is rapidly cleared from the blood. Thus, part of the beneficial pharmacodynamic effects of the drug are perhaps primarily caused by noribogaine in the CNS, as has been reported before (Mash et al., 2000; Zubaran, 2000).

In our 2 h experiment, noribogaine (and its glucuronide, but in lower concentrations) was detected at the earliest time point (0.125 h = 7.5 min), consistent with rapid first-pass metabolism of the parent drug. Maximal concentrations of the metabolites in plasma far exceeded those of the parent compound, as is the case in humans (Mash et al., 2000; Mačiulaitis et al., 2008), and when given intraperitoneally (40 mg/kg) to rats (Baumann et al., 2001). This may be due to a slower clearance rate of noribogaine. Nonetheless, in our study, the difference between the plasma concentrations of ibogaine *versus* its metabolites was more pronounced compared to what has been reported in humans and rats.

In humans, the half-life of noribogaine is considerably longer than that of ibogaine, leading to substantial plasma concentrations of the metabolite long after ibogaine elimination, probably also due to the enterohepatic recirculation of noribogaine (Mash et al., 1995; Litjens and Brunt, 2016; Henstra et al., 2017). In the current study, neither ibogaine nor its metabolites persisted at high levels in either plasma or brain of mice 2 or 8 h following oral administration of ibogaine. Our findings are in accordance with Pearl et al. (1997), where the authors found considerably lower noribogaine levels 19 h after intraperitoneal injection of 40 mg/kg ibogaine in rats compared to the reported persisting high levels of noribogaine in ibogaine-treated patients. This suggests that differences in species, doses, and routes of administration can play an important role for the discrepancies found in rodent *versus* human PK profiles.

To the best of our knowledge, no previous research has reported the *in vivo* single and combined effects of ABCB1/P-gp and ABCG2/BCRP, or OATP1A/1B drug transporters on the plasma pharmacokinetics and tissue disposition of ibogaine and its main metabolites.

Still, Tournier et al. (2009) evaluated the ABCB1- and ABCG2-mediated transport of different compounds including ibogaine labeled with (99 m)Tc-tricarbonyl, in human (h)ABCB1- and hABCG2-transfected HEK293 cells*,* with or without specific inhibitors, and *in situ* by a mouse brain perfusion technique. The *in vitro* transport of ibogaine labeled with (99 m)Tc-tricarbonyl was not influenced by hABCB1or hABCG2, and they also observed that ABCB1- and ABCG2-mediated efflux did not reduce the brain uptake of the radiolabeled ibogaine (Tournier et al., 2009). However, the authors used ibogaine modified with a tricarbonyl core and not the native compound, making it more challenging to compare these findings with ours.

Moreover, Tournier et al. (2010) used *in vitro* ABCB1 and ABCG2 inhibition in flow cytometric assays with hABCB1- and hABCG2-transfected HEK293 cells to study the interaction of ibogaine with these efflux transporters. The authors then demonstrated that both human ABCB1 and ABCG2 were significantly inhibited in a concentration-dependent manner by ibogaine. Nonetheless, the authors did not explore the potential (clinical) implications of these findings.

We observed that the plasma exposure of ibogaine and noribogaine glucuronide was significantly higher in *Abcb1a/1b;Abcg2* ^*−/−*^ mice compared to the WT situation. Although variation was high, the rank order of ibogaine, noribogaine, and the glucuronide AUCs was the same for each compound: WT < *Abcg2* ^*−/−*^ < *Abcb1a/1b;Abcg2* ^*−/−*^ (Figure 2; Table 1). This suggests that the Abcb1a/1b drug efflux transporter plays a more substantial role in limiting the plasma exposure of ibogaine and noribogaine glucuronide.

We consider that the increased plasma exposure of ibogaine and noribogaine glucuronide may be partly explained by a reduced effective pumping (back) of these compounds into the intestinal lumen and/or, for the glucuronide, decreased hepatobiliary excretion in *Abcb1a/1b;Abcg2* ^*−/−*^ compared to wild-type mice. Consistent with this hypothesis, a lower % of dose corrected for the plasma exposure (AUC_0–2h_) of ibogaine and noribogaine glucuronide was found in the SIWC of *Abcb1a/1b;Abcg2* ^*−/−*^ mice compared to the WT situation (Figure 3D; Supplementary Figure S4S). A significantly reduced % of dose of noribogaine glucuronide was also found in the SIWC of single *Abcg2* ^*−/−*^ mice.

However, the liver disposition of noribogaine glucuronide was not detectably increased by Abcb1a/1b and/or Abcg2 deficiency, which seems to refute a marked decrease in biliary excretion. Intrahepatic bile (without the gallbladder) can make up a substantial part of the total liver concentration. Hence, the rapid liver equilibration with blood combined with an increased concentration of noribogaine glucuronide in the hepatocytes of Abcb1a/1b-deficient mice could be offset by a decrease in its biliary concentration, resulting in no substantial alteration in the overall liver disposition.

Furthermore, since judging from the gallbladder data, noribogaine glucuronide was more concentrated in the bile than in the hepatocytes in all strains (Supplementary Figures S4C–F—higher gallbladder-to-plasma ratios *versus* liver-to-plasma ratios), perhaps other drug transporters played a role in concentrating this glucuronide in the bile, such as the ABCC2/MRP2 transporter.

It is known that, both in humans and rodents, ibogaine is excreted not only by the gastrointestinal tract but also via urine (Alper, 2001). We observed a modest increase in the kidney-to-plasma ratio of ibogaine in Abcb1a/1b-deficient mice (Figure 3F), which can be an indicator of the role of mAbcb1a/1b in the excretory transport into urine of this compound.

We further found a significant increase in the brain penetration of ibogaine when mice lack both *Abcb1a/1b* and *Abcg2*, but not in the absence of *Abcg2* (Figure 3B). This then suggests that *Abcb1a/1b* plays a noticeable role in limiting the penetration of ibogaine across the BBB, even though ibogaine presented an intrinsically good brain penetration (brain-to-plasma ratio >3) compared to, for instance, many targeted anti-cancer drugs, which mostly tend to have brain-to-plasma ratios below 0.2 in WT mice. Good brain penetration is, of course, expected for a drug with its main pharmacodynamic targets in the brain, but it does make it harder for an efflux transporter in the BBB to make an impact on the brain accumulation of this compound.

One of the major concerns of ibogaine therapy is the risk of cardiotoxicity that can even lead to the death of patients (Litjens and Brunt, 2016). Interestingly, we observed a slight increase in ibogaine concentration, corrected for the plasma concentration at the time tissues were collected, in the heart of *Abcb1a/1b;Abcg2* ^*−/−*^ mice compared to the other mouse strains (Figure 3H). Hence, at least in mice, Abcb1 in combination with the Abcg2 transporter, which both can be expressed in the heart (Meissner et al., 2002, 2006), might play a role in the susceptibility of the heart to toxicity.

Surprisingly, we did not observe a meaningful impact of the studied drug transporters (and metabolizing enzymes) on noribogaine plasma pharmacokinetics and tissue disposition, despite it being more polar than ibogaine. Still, and in accordance with other PK studies (Staley et al., 1996; Pearl et al., 1997; Zubaran, 1999), we observed that noribogaine readily penetrates the BBB, achieving indeed much higher concentrations in the brain tissue compared with plasma. Moreover, noribogaine distributed somewhat more to the brain than ibogaine (brain-to-plasma ratios of 7.2 *versus* 3.4 in WT mice, respectively, and 8.3 *versus* 5.2 in *Abcb1a/1b;Abcg2* ^*−/−*^ mice). This could have pharmacodynamic implications since, in absolute terms, there is far more noribogaine present in the (mouse) circulation than the parent compound. This ties in very well with noribogaine being a major active metabolite, also contributing to the therapeutic effect of ibogaine.

In humans, several ABCB1 single nucleotide polymorphisms (SNPs) have been identified, and some of them have been associated with low ABCB1 function (Lin and Yamazaki, 2003; Ho and Kim, 2005; Fung and Gottesman, 2009). Moreover, nonsense mutations associated with a total loss of function of ABCB1 can also occur in humans, albeit rarely (Baudou et al., 2020). The same applies to the ABCG2 transporter, where several detrimental SNPs in the *ABCG2* gene and even full gene deficiency have been identified (Kondo et al., 2004; Tamura et al., 2006; Toyoda et al., 2021). Thus, these genetic variants in ABCB1 and ABCG2 can be a determinant of interindividual variability in drug response. The results reported so far can then be relevant in the context of individuals having low ABCB1/ABG2 activity due to polymorphisms and being exposed concurrently to drugs (frequently used in combination), food contaminants, and natural food components that can modulate ABCB1/ABCG2 activity. However, as the overall absolute impact of these transporters on ibogaine and noribogaine pharmacokinetics appears to be modest, at least in mice, the associated risk in humans might be mild.

Our findings suggest that the human OATP1B1/1B3 transporters do not play an important role in controlling clearance and disposition of ibogaine and its metabolites, as no meaningful differences were found in the plasma exposure and liver uptake of those compounds in our transgenic mouse strains. Nevertheless, in the absence of mOatp1a/1b, we observed a substantial decrease in the liver accumulation of noribogaine and noribogaine glucuronide. For ibogaine itself, most likely mOatp1a/1b also plays a role in the liver uptake and thus clearance of ibogaine, but the experimental variation in the *Oatp1a/1b* ^*−/−*^ mice obscured this effect.

As van de Steeg et al. (2010) detected mRNA of Slco1a4 and Slco1a6 in the small intestine of mice, and Sugiura et al. (2010) revealed by immunohistochemistry the presence of mOatp1a in the apical membrane of intestinal epithelial cells in WT mice, we also evaluated the possible role of Oatp1a in the intestinal absorption of ibogaine and its metabolites. Our results indicate that mOatp1a transporters are not instrumental in the intestinal absorption of ibogaine, but they do contribute to noribogaine and noribogaine glucuronide SIWC disposition, perhaps reflecting the reduced liver accumulation of the metabolites.

Up until now, there is no publicly available evidence on whether ibogaine is a significant *in vivo* CYP3A substrate or not. The data obtained in our 8 h oral experiment suggest that CYP3A is not a main player in metabolizing ibogaine in mice. Still, we observed a noticeable increase in the absolute concentrations of ibogaine and noribogaine in the brain of *Cyp3a* ^*−/−*^ and especially Cyp3aXAV mice, suggesting that changes in plasma clearance of these compounds may have a disproportionate impact on their pharmacodynamic effects. Something similar might apply to the potentially toxic effects of ibogaine/noribogaine in the heart. It may thus be important to try and control and monitor the plasma exposure of ibogaine and noribogaine carefully in patients in order to minimize the risk of over-or underexposure. Previous literature reported a minor contribution of human CYP2C19 in ibogaine metabolism. Therefore, we cannot exclude that the up-regulation of mCyp2c enzymes in *Cyp3a* ^*−/−*^ mice, as described before (van Waterschoot et al., 2008), may also have contributed to the metabolism of ibogaine. This could then potentially mask a more noticeable impact of CYP3A on ibogaine pharmacokinetics than suggested by our experiments in mice.

Of note, for ethical and practical reasons, we used only female mice in our studies. Pearl et al. (1997) showed that ibogaine produced more robust pharmacodynamic effects in female than in male rats. Perhaps this could be due to higher levels of ibogaine in the brain and plasma of female rats. Thus, future studies in male mice might be of interest, and also to assess if potential sex/gender differences in pharmacokinetics, safety, and efficacy of ibogaine/noribogaine also apply to humans. Moreover, using a higher dose of ibogaine in mice might be relevant, especially when addressing ibogaine pharmacodynamics, as 10 mg/kg in mice is relatively low since physiological processes/metabolic rates in rodents are faster than in humans (Nair and Jacob, 2016). Another limitation of our study is that we did not use a single knockout strain for the Abcb1a/1b efflux transporter. This was because we did not observe a strong impact of the Abcb1a/1b; Abcg2 combination knockout mice on the disposition of ibogaine and its metabolites. We had included the Abcg2 knockout mouse strain because we considered that noribogaine glucuronide could be a substrate of mouse Abcg2 as it has been shown that ABCG2 substrates include, among others, glucuronide conjugates (Mao and Unadkat, 2015). Moreover, comparison between the *Abcg2* ^*−/−*^ and *Abcb1a/1b;Abcg2* ^*−/−*^ mice could reveal any Abcb1a/1b effects on the disposition of ibogaine and its metabolites.

## Conclusion

This study shows that ABCB1 (P-gp), together with ABCG2 (BCRP), can modestly restrict the oral availability and brain penetration of ibogaine. Therefore, genetic polymorphisms and/or pharmacologic inhibition of these transporters might impact the safety and therapy response of ibogaine. Coadministration of ABCB1/ABCG2 inhibitors may be a possibility to further enhance ibogaine exposure in patients, particularly in the brain, which could lead to even more long-lasting pharmacodynamic outcomes. Given the overall modest impact of these ABC transporters, however, dramatic shifts are unlikely. Nevertheless, additional studies may address any potentially altered toxicity that could occur by using this pharmacological approach. Human OATP transporters and CYP3A4 enzyme have a limited impact (if any) on restricting the plasma exposure and tissue disposition of ibogaine and its metabolites, at least in mice. This may be considered beneficial for the therapeutic application of ibogaine as no substantial risk of high variation in bioavailability due to variable OATP/CYP3A4 activity is anticipated. Nonetheless, we cannot exclude that compensatory changes in other ibogaine-detoxifying systems in the OATP/CYP3A genetically modified mouse strains had occurred; thus, further preclinical studies are warranted. Likewise, further research should be conducted to determine the role of ibogaine/noribogaine as modulator (i.e., inducer or inhibitor) of the studied proteins to better predict clinically significant drug interactions during ibogaine therapy.

## Data Availability

The raw data supporting the conclusions of this article will be made available by the authors, without undue reservation.
