# Supplementary material for: Drug Transporters ABCB1 (P-gp) and OATP, but not Drug-Metabolizing Enzyme CYP3A4, Affect the Pharmacokinetics of the Psychoactive Alkaloid Ibogaine and its Metabolites
Source: Front Pharmacol. 2022 Mar 4;13:855000. doi: 10.3389/fphar.2022.855000 (PMC8931498; doi:10.3389/fphar.2022.855000)
Supplement: Supplementary file 1 [file Presentation1.pdf]

Supplementary Material

1 Supplementary Figures and Tables

1. Supplementary Figures

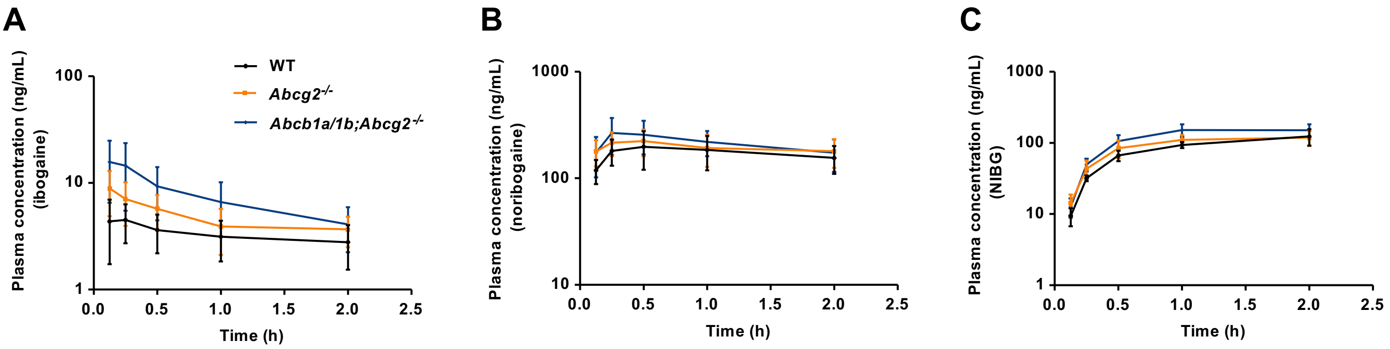

Supplementary Figure S1. Semi-log plasma concentration-time curves of ibogaine **(A)**, noribogaine **(B)**, and noribogaine glucuronide (NIBG) **(C)** over 2 h in female wild-type (WT), *Abcg2*$^{-/-}$, and *Abcb1a/1b;Abcg2*$^{-/-}$ mice after oral administration of 10 mg/kg ibogaine (n = 7). Data are presented as mean ± SD.

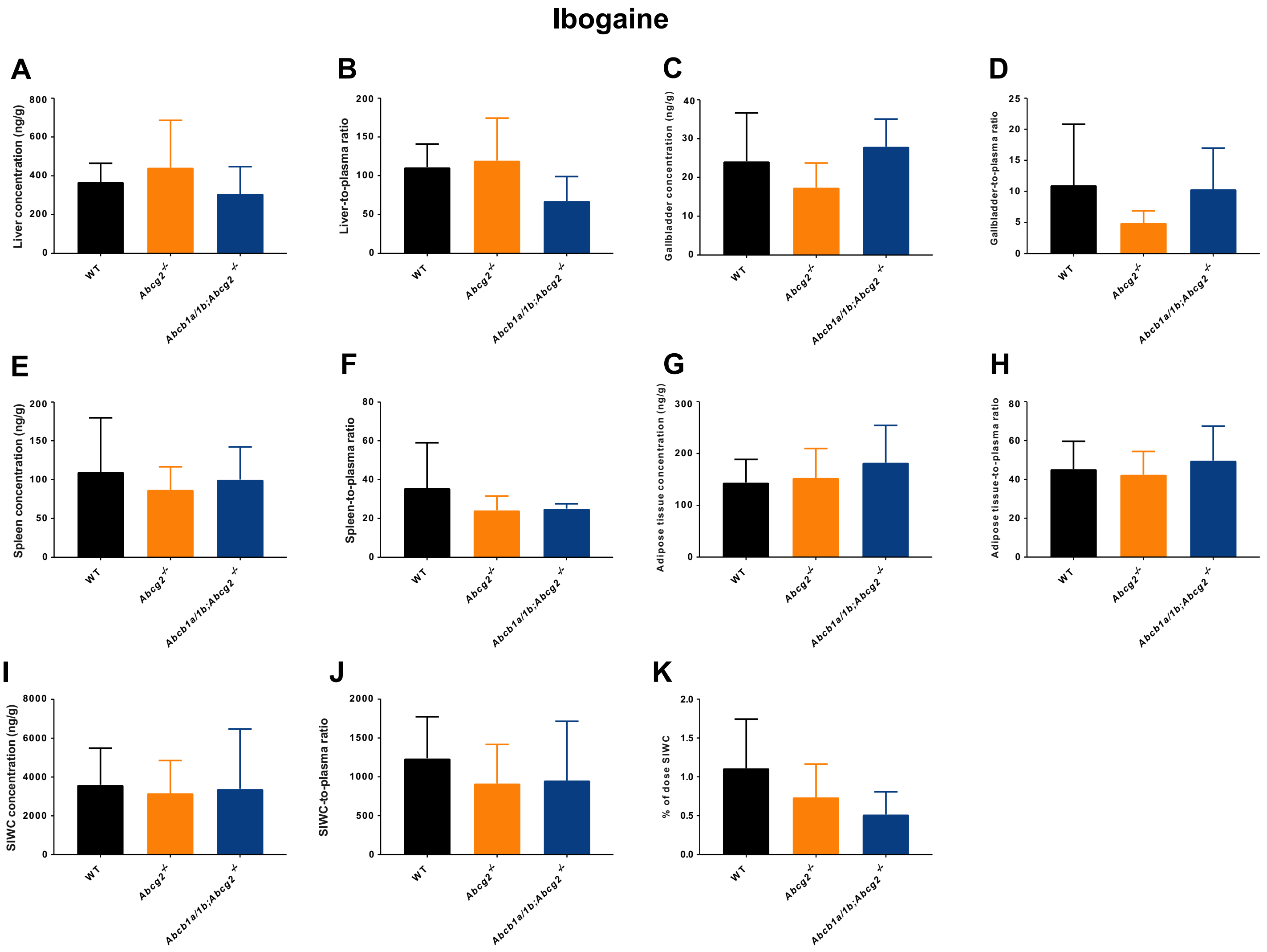

Supplementary Figure S2. Ibogaine tissue concentration **(A, C, E, G, I)**, tissue-to-plasma ratio **(B, D, F, H, J)**, and small intestine together with the fecal content (SIWC) as percentage of dose **(K)** in female wild-type (WT), *Abcg2*$^{-/-}$, and *Abcb1a/1b;Abcg2*$^{-/-}$ mice, over 2 h after oral administration of 10 mg/kg ibogaine (n = 6-7). Data are presented as mean ± SD.

Noribogaine

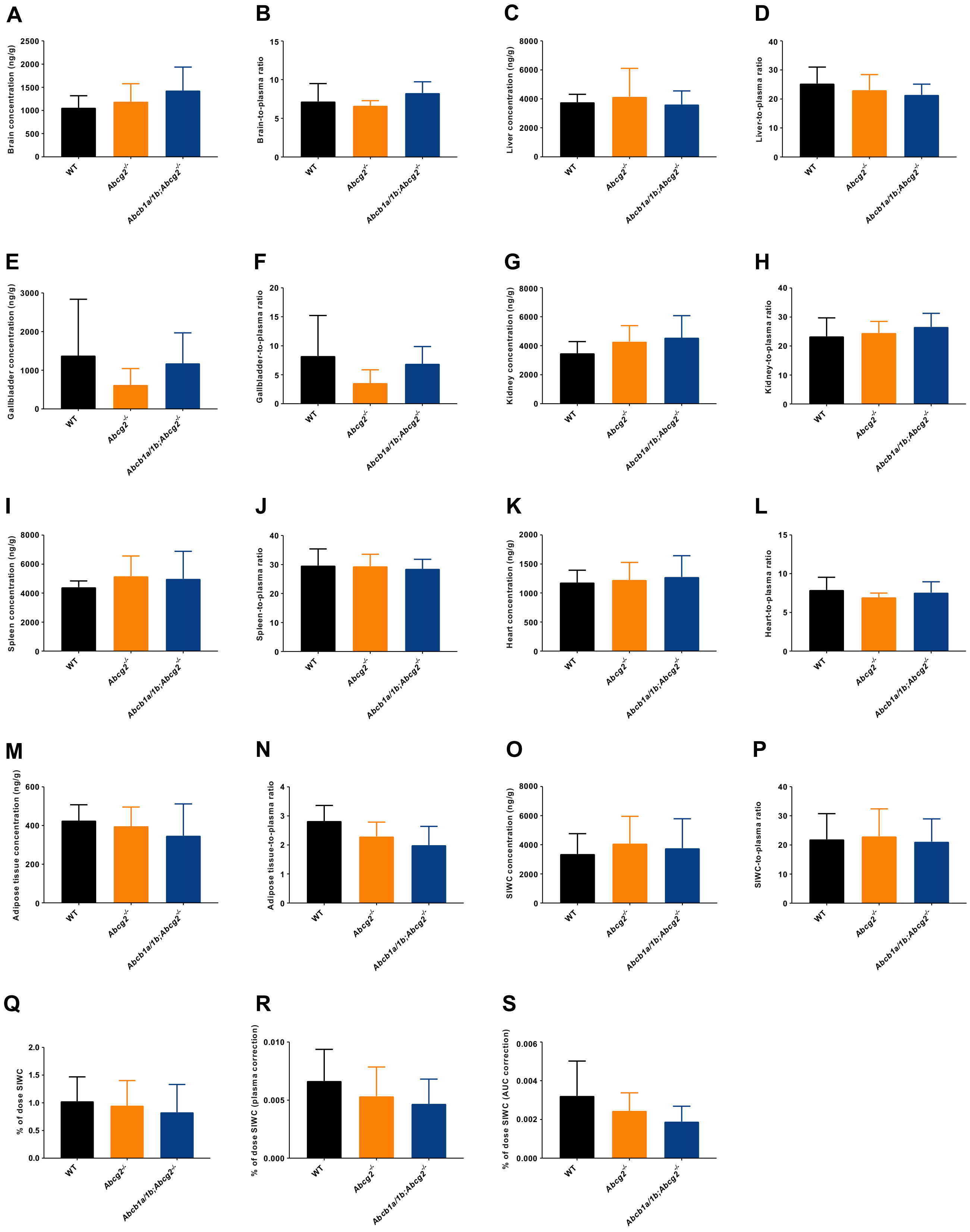

Supplementary Figure S3. Noribogaine tissue concentration **(A, C, E, G, I, K, M, O)**, tissue-to-plasma ratio **(B, D, F, H, J, L, N, P)**, and small intestine together with the fecal content (SIWC) as percentage of dose **(Q)** corrected for the plasma concentration at 2 h **(R)** and corrected for the area under the curve (AUC) **(S)** in female wild-type (WT), *Abcg2*$^{-/-}$, and *Abcb1a/1b;Abcg2*$^{-/-}$ mice over 2 h after oral administration of 10 mg/kg ibogaine (n = 6-7). Data are presented as mean ± SD.

Noribogaine glucuronide

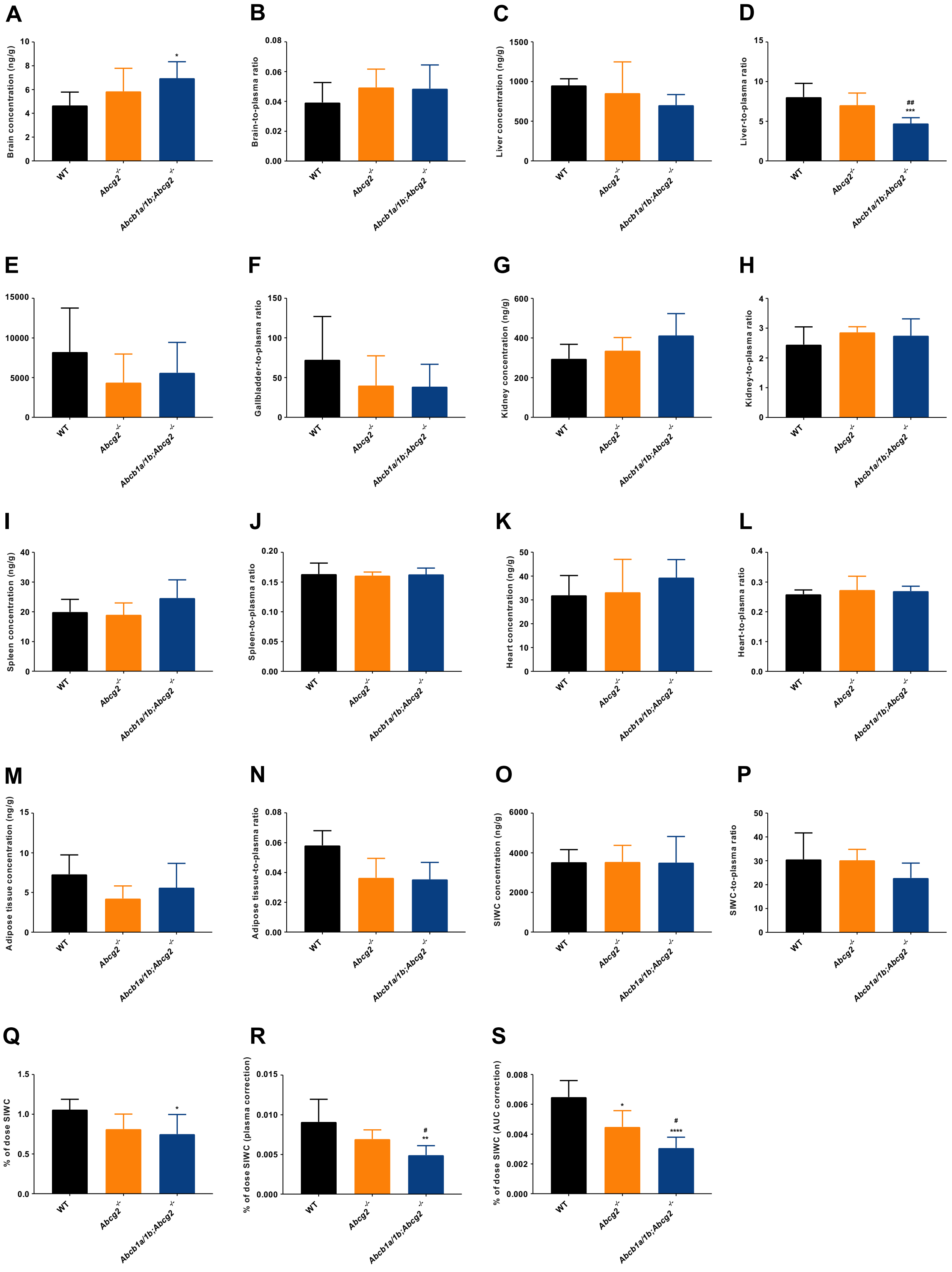

Supplementary Figure S4. Noribogaine glucuronide tissue concentration **(A, C, E, G, I, K, M, O)**, tissue-to-plasma ratio **(B, D, F, H, J, L, N, P)**, and small intestine together with the fecal content (SIWC) as percentage of dose **(Q)** corrected for the plasma concentration at 2 h **(R)** and corrected for the area under the curve (AUC) **(S)** in female wild-type (WT), *Abcg2*$^{-/-}$, and *Abcb1a/1b;Abcg2*$^{-/-}$ mice over 2 h after oral administration of 10 mg/kg ibogaine (n = 6-7). SIWC, small intestine together with the fecal content. Data are presented as mean ± SD. *, P <0.05; **, P < 0.01; ***, P < 0.001; ****, P < 0.0001 compared to wild-type mice; $^{\#}$, P < 0.05; $^{\#\#}$, P < 0.01 comparing *Abcb1a/1b;Abcg2*$^{-/-}$ with *Abcg2*$^{-/-}$mice.

Ibogaine

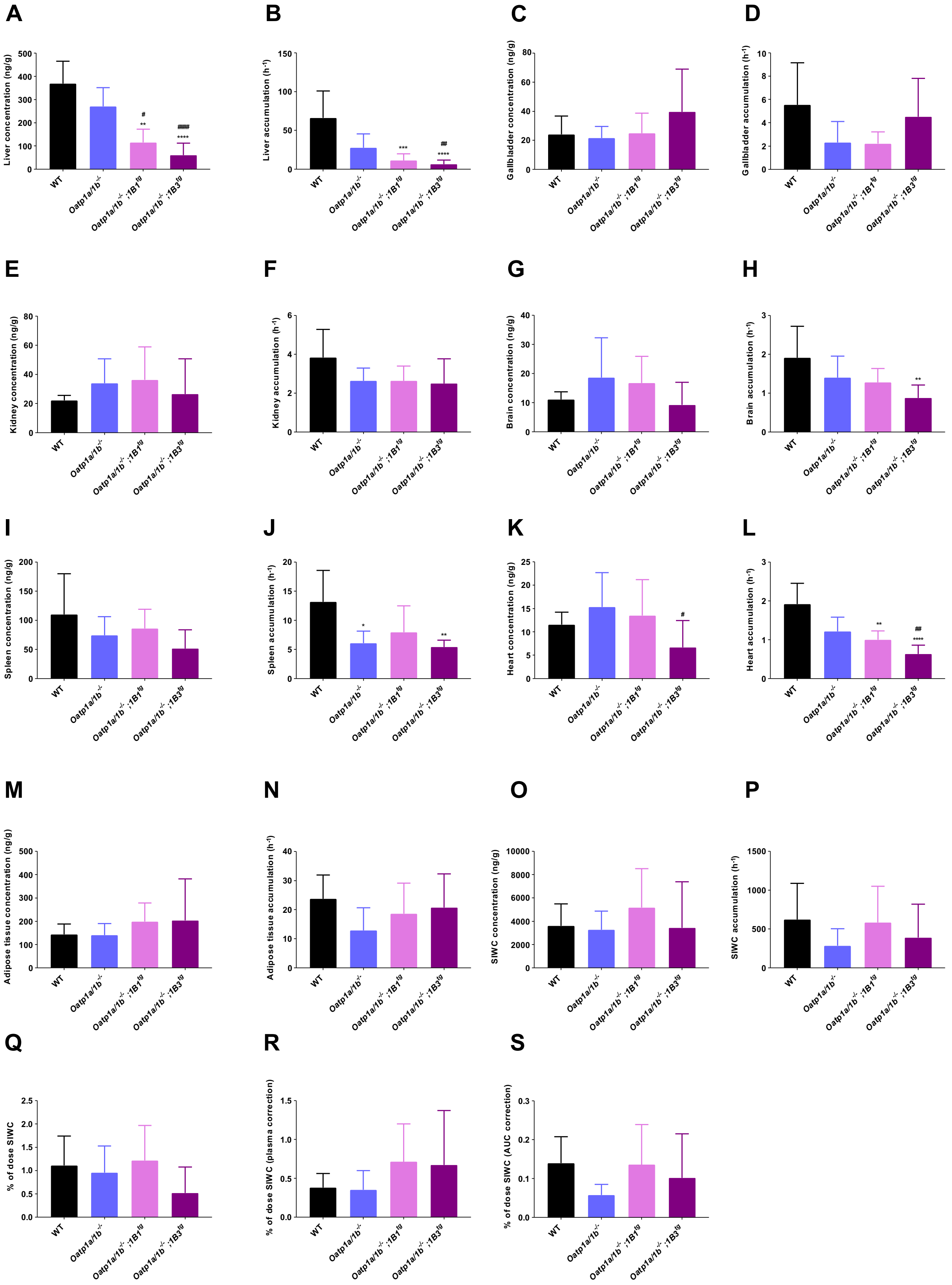

Supplementary Figure S5. Ibogaine tissue concentration **(A, C, E, G, I, K, M, O)**, tissue accumulation **(B, D, F, H, J, L, N, P)** and small intestine together with the fecal content (SIWC) as percentage (%) of dose without **(Q)** or with corrections (for the plasma concentration at 2 h **(R)** or for the plasma AUC **(S)**), in female wild-type (WT), *Oatp1a/1b*$^{-/-}$, *Oatp1a/1b*$^{-/-}$*;1B1*^{tg}, and *Oatp1a/1b*$^{-/-}$*;1B3*^{tg} mice, over 2 h after oral administration of 10 mg/kg ibogaine (n = 6-7). Data are presented as mean ± SD. *, $P < 0.05$; **, $P < 0.01$; ***, $P < 0.001$; ****, $P < 0.0001$ compared to wild-type mice; $^{\#}$, $P < 0.05$; $^{\#\#}$, $P < 0.01$; $^{\#\#\#}$, $P < 0.001$; $^{\#\#\#\#}$, $P < 0.0001$ comparing *Oatp1a/1b*$^{-/-}$*;1B1*^{tg} or *Oatp1a/1b*$^{-/-}$*;1B3*^{tg} with *Oatp1a/1b*$^{-/-}$ mice.

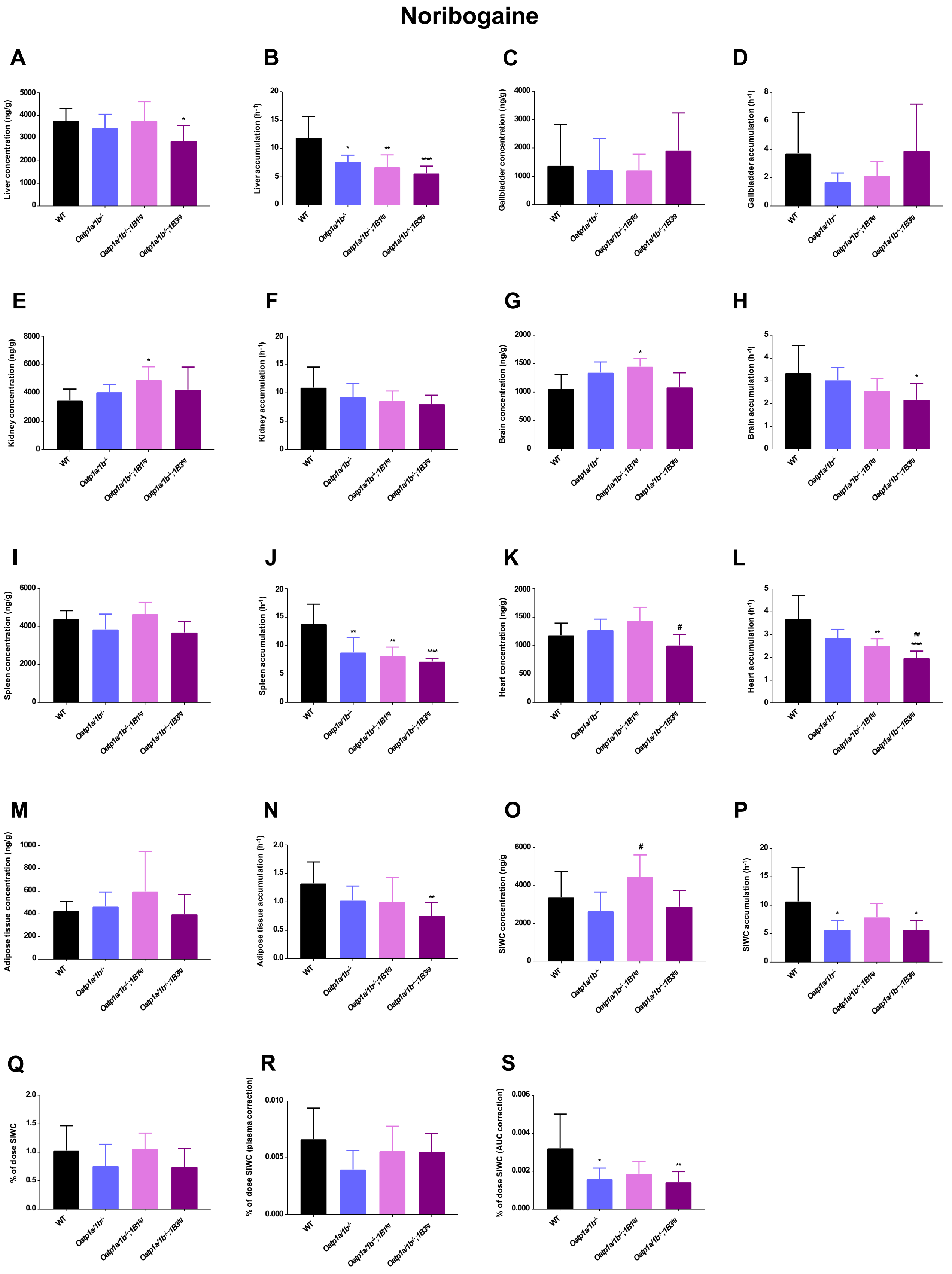

Supplementary Figure S6. Noribogaine tissue concentration **(A, C, E, G, I, K, M, O)**, tissue accumulation **(B, D, F, H, J, L, N, P)** and small intestine together with the fecal content (SIWC) as percentage (%) of dose without **(Q)** or with corrections (for the plasma concentration at 2 h **(R)** or for the plasma AUC **(S)**), in female wild-type (WT), *Oatp1a/1b*$^{-/-}$, *Oatp1a/1b*$^{-/-}$*;1B1*^{tg}, and *Oatp1a/1b*$^{-/-}$*;1B3*^{tg} mice, over 2 h after oral administration of 10 mg/kg ibogaine (n = 7). Data are presented as mean ± SD. *, $P < 0.05$; **, $P < 0.01$; ****, $P < 0.0001$ compared to wild-type mice; $^{\#}$, $P < 0.05$; $^{\#\#}$, $P < 0.01$; comparing *Oatp1a/1b*$^{-/-}$*;1B1*^{tg} or *Oatp1a/1b*$^{-/-}$*;1B3*^{tg} with *Oatp1a/1b*$^{-/-}$ mice.

Noribogaine glucuronide

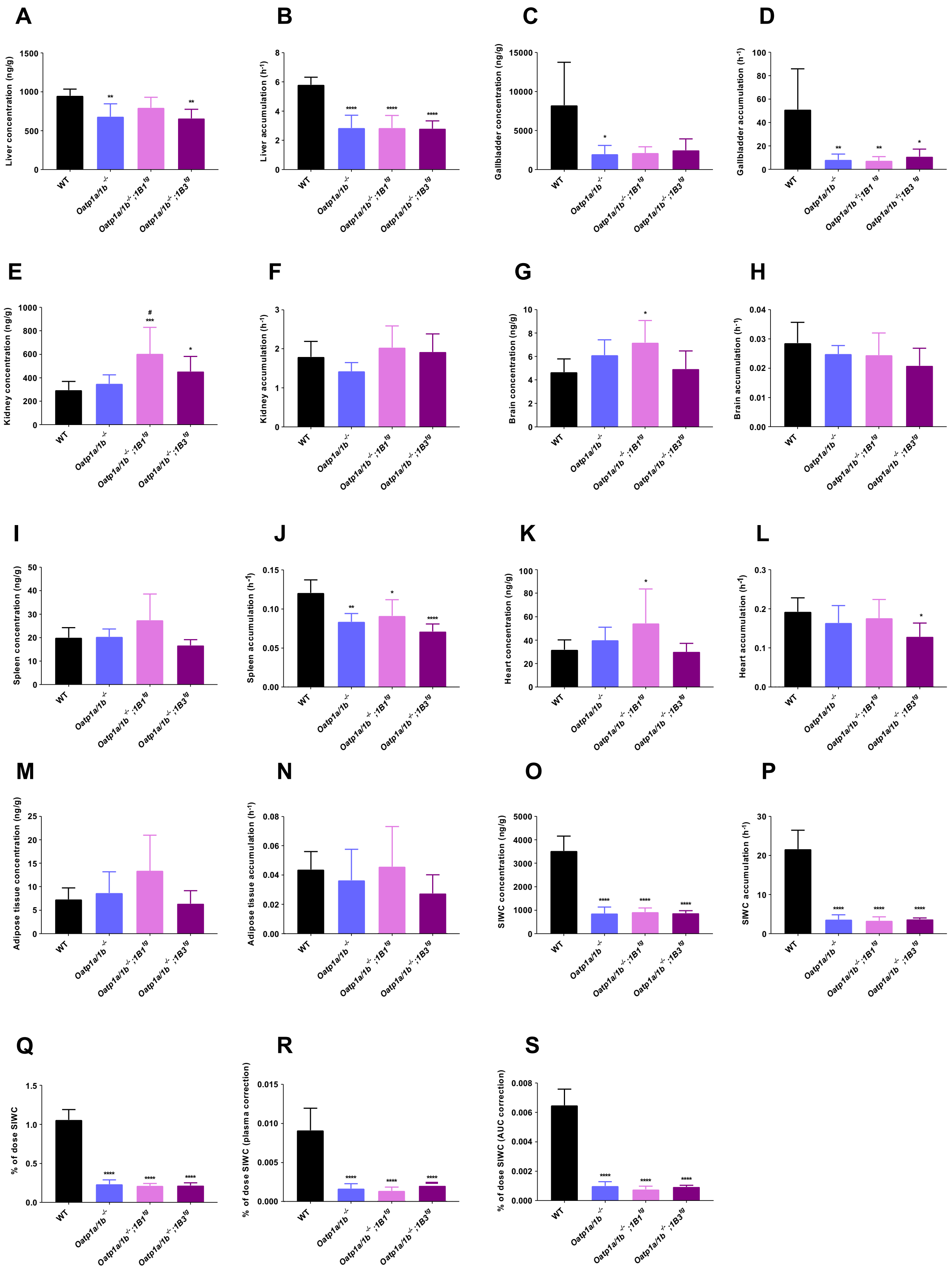

Supplementary Figure S7. Noribogaine glucuronide tissue concentration **(A, C, E, G, I, K, M, O)**, tissue accumulation **(B, D, F, H, J, L, N, P)** and small intestine together with the fecal content (SIWC) as percentage (%) of dose without **(Q)** or with corrections (for the plasma concentration at 2 h **(R)** or for the plasma AUC **(S)**), in female wild-type (WT), *Oatp1a/1b*$^{-/-}$, *Oatp1a/1b*$^{-/-}$*;1B1*^{tg}, and *Oatp1a/1b*$^{-/-}$*;1B3*^{tg} mice, over 2 h after oral administration of 10 mg/kg ibogaine (n = 6-7). Data are presented as mean ± SD. *, $P < 0.05$; **, $P < 0.01$; ***, $P < 0.001$; ****, $P < 0.0001$ compared to wild-type mice; #, $P < 0.05$; ###, $P < 0.001$ comparing *Oatp1a/1b*$^{-/-}$*;1B1*^{tg} or *Oatp1a/1b*$^{-/-}$*;1B3*^{tg} with *Oatp1a/1b*$^{-/-}$ mice.

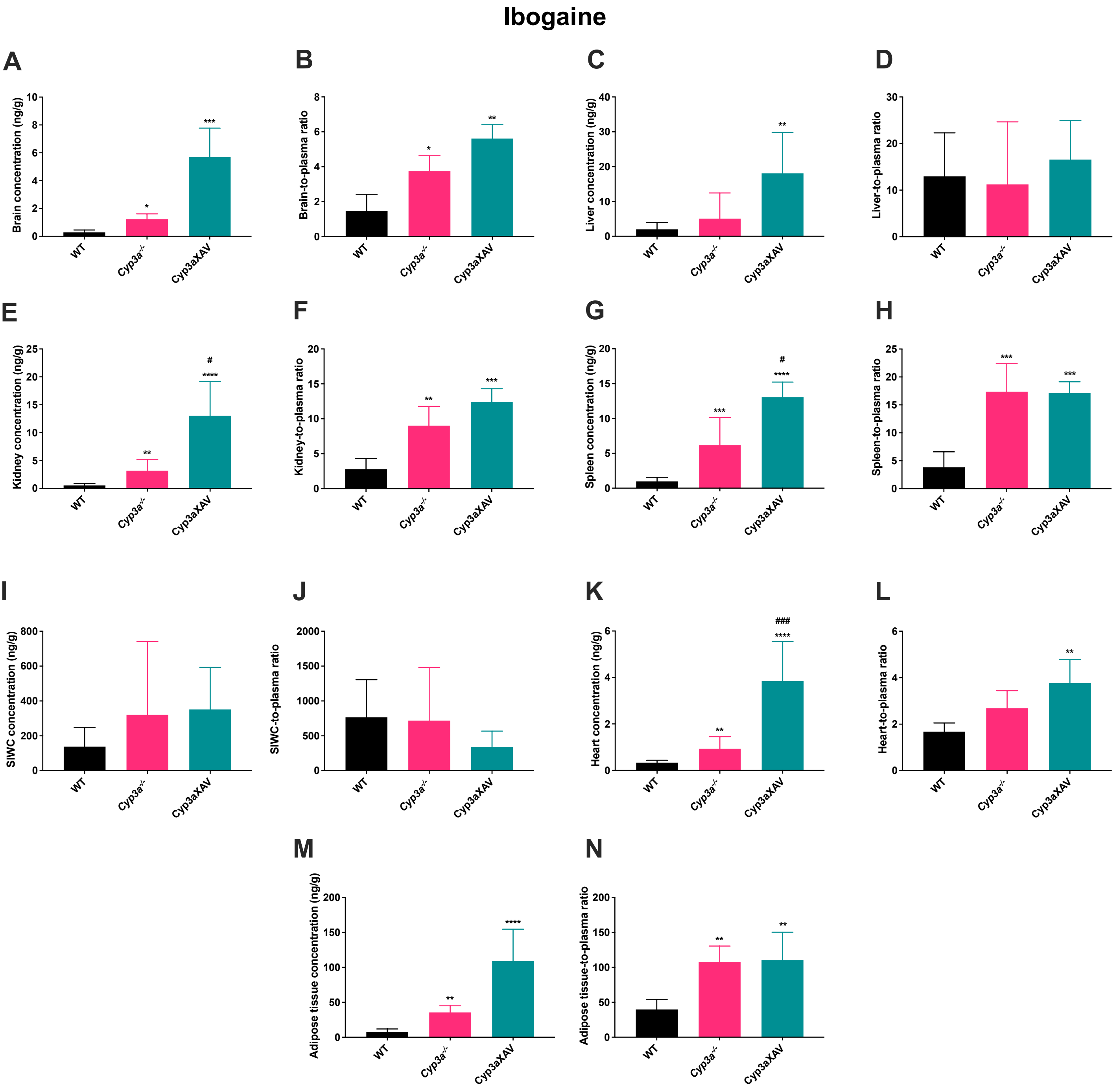

Supplementary Figure S8. Ibogaine tissue concentration **(A, C, E, G, I, K, M)** and tissue-to-plasma ratio **(B, D, F, H, J, L, N)** in female wild-type (WT), Cyp3a knockout (*Cyp3a*$^{-/-}$), and *Cyp3a*$^{-/-}$ mice with specific transgenic expression of human CYP3A4 in liver and intestine (Cyp3aXAV), over 8 h after oral administration of 10 mg/kg ibogaine (n = 3-6). SIWC, small intestine together with the fecal content. Data are presented as mean ± SD. *, $P < 0.05$; **, $P < 0.01$; ***, $P < 0.001$; ****, $P < 0.0001$ compared to wild-type mice; $^{\#}$, $P < 0.05$; $^{\#\#\#}$, $P < 0.001$ comparing Cyp3aXAV with *Cyp3a*$^{-/-}$ mice.

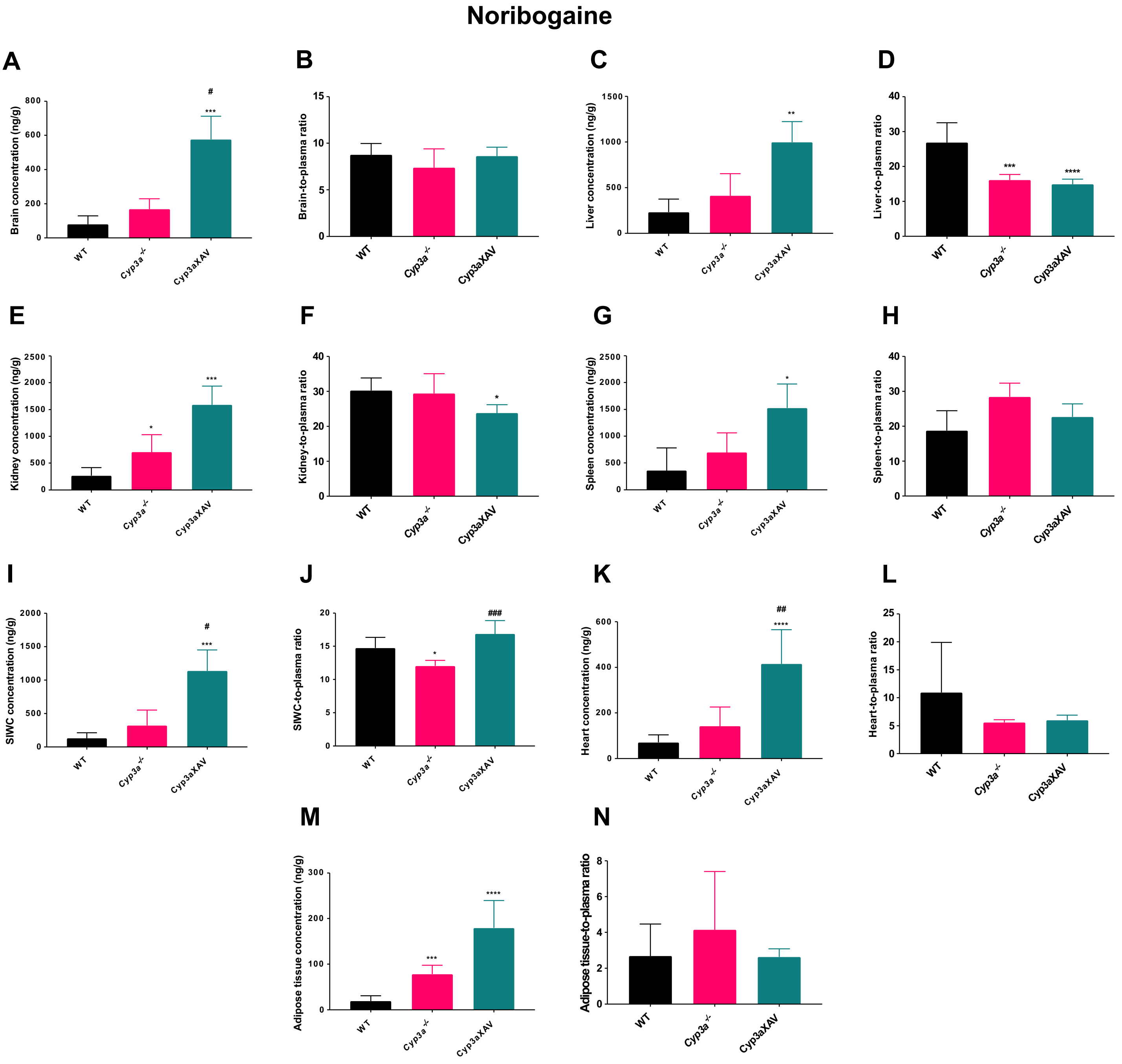

Supplementary Figure S9. Noribogaine tissue concentration **(A, C, E, G, I, K, M)** and tissue-to-plasma ratio **(B, D, F, H, J, L, N)** in female wild-type (WT), Cyp3a knockout (*Cyp3a*$^{-/-}$), and *Cyp3a*$^{-/-}$ mice with specific transgenic expression of human CYP3A4 in liver and intestine (Cyp3aXAV), over 8 h after oral administration of 10 mg/kg ibogaine (n = 4-6). SIWC, small intestine together with the fecal content. Data are presented as mean ± SD. *, $P < 0.05$; **, $P < 0.01$; ***, $P < 0.001$; ****, $P < 0.0001$ compared to wild-type mice; #, $P < 0.05$; ##, $P < 0.01$; ###, $P < 0.001$ comparing Cyp3aXAV with *Cyp3a*$^{-/-}$ mice.

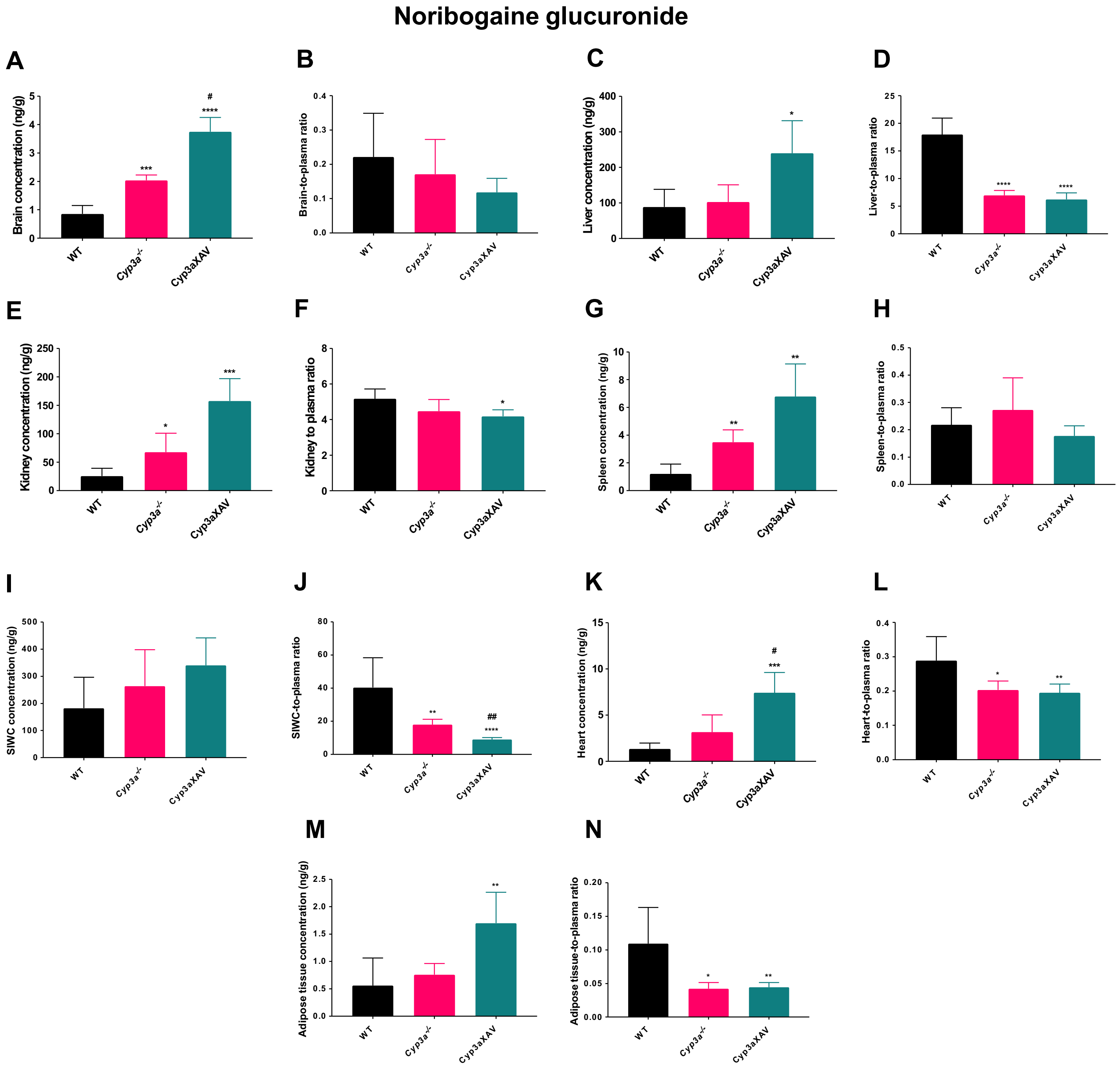

Supplementary Figure S10. Noribogaine glucuronide (NIBG) tissue concentration **(A, C, E, G, I, K, M)** and tissue-to-plasma ratio **(B, D, F, H, J, L, N)** in female wild-type (WT), Cyp3a knockout (*Cyp3a*$^{-/-}$), and *Cyp3a*$^{-/-}$ mice with specific transgenic expression of human CYP3A4 in liver and intestine (Cyp3aXAV), over 8 h after oral administration of 10 mg/kg ibogaine (n = 4-6). SIWC, small intestine together with the fecal content. Data are presented as mean ± SD. *, $P < 0.05$; **, $P < 0.01$; ***, $P < 0.001$; ****, $P < 0.0001$ compared to wild-type mice; $^{\#}$, $P < 0.05$; $^{\#\#}$, $P < 0.01$ comparing Cyp3aXAV with *Cyp3a*$^{-/-}$ mice.

1.2 Supplementary Table

Table S1: Plasma pharmacokinetic (PK) parameters of ibogaine, noribogaine, and noribogaine glucuronide 8 h after oral administration of 10 mg/kg ibogaine to female wild-type, *Cyp3a*$^{-/-}$ and Cyp3aXAV mice. [a]

Compound and PK Parameter	Genotype		
	**Wild-type**	***Cyp3a***$^{-/-}$	**Cyp3aXAV**
**Ibogaine**			
$AUC_{0\text{-}8h}$ (h*ng/mL)	13.5 ± 4.5	18.5 ± 2.6	31.4 ± 12.5**
Fold increase $AUC_{0\text{-}8h}$	1.0	1.4	2.3
C_{max}, ng/mL	6.46 ± 3.72	6.59 ± 0.89	12.01 ± 5.14
T_{max}, h	0.25	0.25	0.25
**Noribogaine**			
$AUC_{0\text{-}8h}$ (h*ng/mL)	655 ± 220	1073 ± 320*	1400 ± 320***
Fold increase $AUC_{0\text{-}8h}$	1.0	1.6	2.1
C_{max}, ng/mL	217 ± 37	280 ± 75	328 ± 75*
T_{max}, h	0.50 (0.50 – 1)	0.50 (0.50 – 1)	0.50 (0.50 – 1)
**Noribogaine glucuronide**			
$AUC_{0\text{-}8h}$ (h*ng/mL)	219 ± 50	422 ± 110**	536 ± 117****
Fold increase $AUC_{0\text{-}8h}$	1.0	2.0	2.4
C_{max}, ng/mL	70.0 ± 13.6	114 ± 24**	123 ± 28**
T_{max}, h	1	1	1 (1 – 2)

[a]Data are presented as mean ± SD (n = 4-6), except for T_{max} where median (range) is presented. $AUC_{0\text{-}8h}$, area under the plasma concentration-time curve from zero to 8 hours; C_{max}, maximum concentration in plasma; T_{max}, time point (h) of maximum plasma concentration. *, $P < 0.05$; **, $P < 0.01$; ***, $P < 0.001$; ****, $P < 0.0001$ compared to wild-type mice.
